# Cyber risk and cybersecurity: a systematic review of data availability

**DOI:** 10.1057/s41288-022-00266-6

**Published:** 2022-02-17

**Authors:** Frank Cremer, Barry Sheehan, Michael Fortmann, Arash N. Kia, Martin Mullins, Finbarr Murphy, Stefan Materne

**Affiliations:** 1 University of Limerick, Limerick, Ireland; 2 TH Köln University of Applied Sciences, Cologne, Germany

**Keywords:** Cyber insurance, Cyber risk, Open data, Systematic review, Cybersecurity

## Abstract

Cybercrime is estimated to have cost the global economy just under USD 1 trillion in 2020, indicating an increase of more than 50% since 2018. With the average cyber insurance claim rising from USD 145,000 in 2019 to USD 359,000 in 2020, there is a growing necessity for better cyber information sources, standardised databases, mandatory reporting and public awareness. This research analyses the extant academic and industry literature on cybersecurity and cyber risk management with a particular focus on data availability. From a preliminary search resulting in 5219 cyber peer-reviewed studies, the application of the systematic methodology resulted in 79 unique datasets. We posit that the lack of available data on cyber risk poses a serious problem for stakeholders seeking to tackle this issue. In particular, we identify a lacuna in open databases that undermine collective endeavours to better manage this set of risks. The resulting data evaluation and categorisation will support cybersecurity researchers and the insurance industry in their efforts to comprehend, metricise and manage cyber risks.

**Supplementary Information:**

The online version contains supplementary material available at 10.1057/s41288-022-00266-6.

## Introduction

Globalisation, digitalisation and smart technologies have escalated the propensity and severity of cybercrime. Whilst it is an emerging field of research and industry, the importance of robust cybersecurity defence systems has been highlighted at the corporate, national and supranational levels. The impacts of inadequate cybersecurity are estimated to have cost the global economy USD 945 billion in 2020 (Maleks Smith et al. 2020). Cyber vulnerabilities pose significant corporate risks, including business interruption, breach of privacy and financial losses (Sheehan et al. 2019). Despite the increasing relevance for the international economy, the availability of data on cyber risks remains limited. The reasons for this are many. Firstly, it is an emerging and evolving risk; therefore, historical data sources are limited (Biener et al. 2015). It could also be due to the fact that, in general, institutions that have been hacked do not publish the incidents (Eling and Schnell 2016). The lack of data poses challenges for many areas, such as research, risk management and cybersecurity (Falco et al. 2019). The importance of this topic is demonstrated by the announcement of the European Council in April 2021 that a centre of excellence for cybersecurity will be established to pool investments in research, technology and industrial development. The goal of this centre is to increase the security of the internet and other critical network and information systems (European Council 2021).

This research takes a risk management perspective, focusing on cyber risk and considering the role of cybersecurity and cyber insurance in risk mitigation and risk transfer. The study reviews the existing literature and open data sources related to cybersecurity and cyber risk. This is the first systematic review of data availability in the general context of cyber risk and cybersecurity. By identifying and critically analysing the available datasets, this paper supports the research community by aggregating, summarising and categorising all available open datasets. In addition, further information on datasets is attached to provide deeper insights and support stakeholders engaged in cyber risk control and cybersecurity. Finally, this research paper highlights the need for open access to cyber-specific data, without price or permission barriers.

**Fig. 1:**
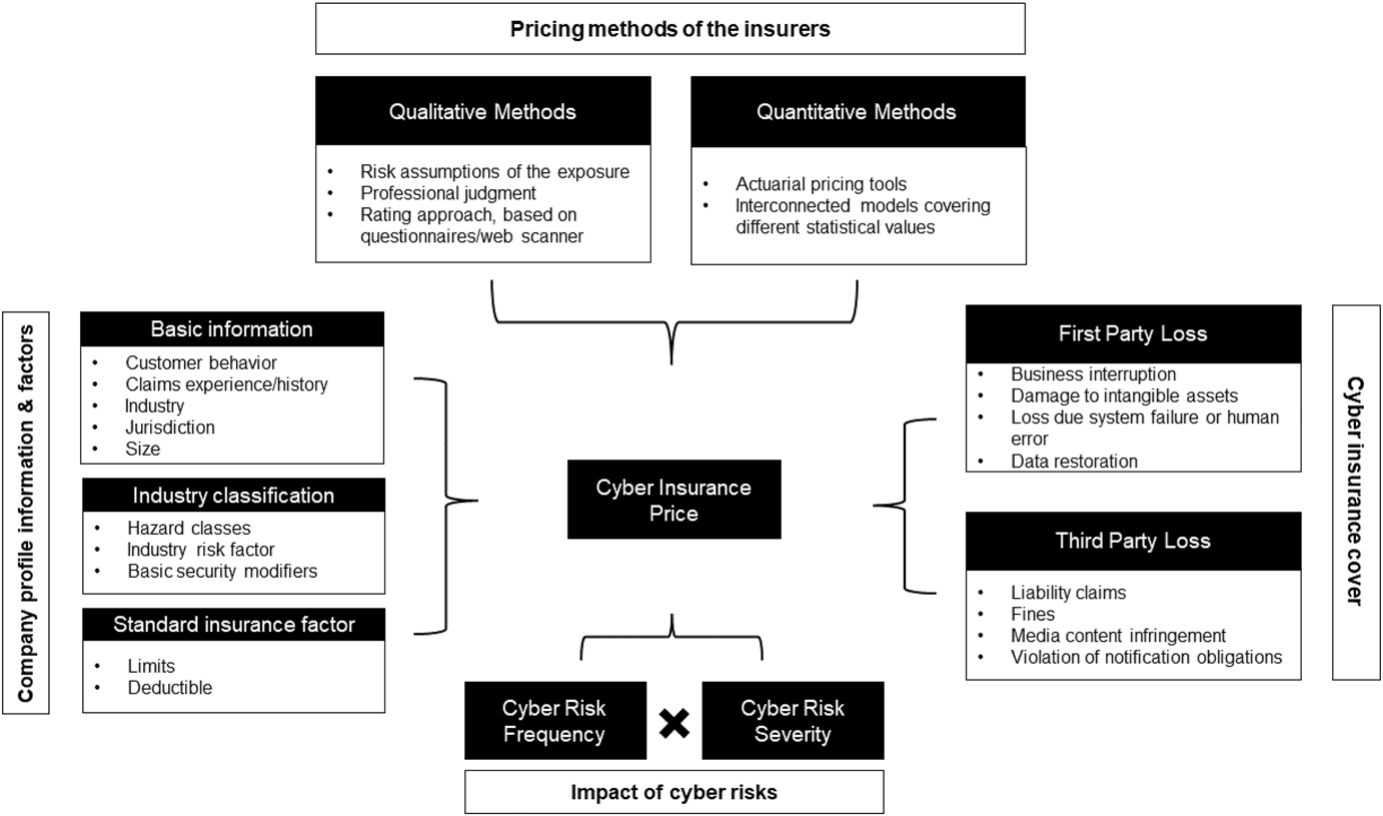
An overview of the current cyber insurance informational and methodological landscape, adapted from EIOPA (2018) and Romanosky et al. (2019)

The identified open data can support cyber insurers in their efforts on sustainable product development. To date, traditional risk assessment methods have been untenable for insurance companies due to the absence of historical claims data (Sheehan et al. 2021). These high levels of uncertainty mean that cyber insurers are more inclined to overprice cyber risk cover (Kshetri 2018). Combining external data with insurance portfolio data therefore seems to be essential to improve the evaluation of the risk and thus lead to risk-adjusted pricing (Bessy-Roland et al. 2021). This argument is also supported by the fact that some re/insurers reported that they are working to improve their cyber pricing models (e.g. by creating or purchasing databases from external providers) (EIOPA 2018). Figure 1 provides an overview of pricing tools and factors considered in the estimation of cyber insurance based on the findings of EIOPA (2018) and the research of Romanosky et al. (2019). The term cyber risk refers to all cyber risks and their potential impact.

Besides the advantage of risk-adjusted pricing, the availability of open datasets helps companies benchmark their internal cyber posture and cybersecurity measures. The research can also help to improve risk awareness and corporate behaviour. Many companies still underestimate their cyber risk (Leong and Chen 2020). For policymakers, this research offers starting points for a comprehensive recording of cyber risks. Although in many countries, companies are obliged to report data breaches to the respective supervisory authority, this information is usually not accessible to the research community. Furthermore, the economic impact of these breaches is usually unclear.

As well as the cyber risk management community, this research also supports cybersecurity stakeholders. Researchers are provided with an up-to-date, peer-reviewed literature of available datasets showing where these datasets have been used. For example, this includes datasets that have been used to evaluate the effectiveness of countermeasures in simulated cyberattacks or to test intrusion detection systems. This reduces a time-consuming search for suitable datasets and ensures a comprehensive review of those available. Through the dataset descriptions, researchers and industry stakeholders can compare and select the most suitable datasets for their purposes. In addition, it is possible to combine the datasets from one source in the context of cybersecurity or cyber risk. This supports efficient and timely progress in cyber risk research and is beneficial given the dynamic nature of cyber risks.

Cyber risks are defined as “operational risks to information and technology assets that have consequences affecting the confidentiality, availability, and/or integrity of information or information systems” (Cebula et al. 2014). Prominent cyber risk events include data breaches and cyberattacks (Agrafiotis et al. 2018). The increasing exposure and potential impact of cyber risk have been highlighted in recent industry reports (e.g. Allianz 2021; World Economic Forum 2020). Cyberattacks on critical infrastructures are ranked 5th in the World Economic Forum's Global Risk Report. Ransomware, malware and distributed denial-of-service (DDoS) are examples of the evolving modes of a cyberattack. One example is the ransomware attack on the Colonial Pipeline, which shut down the 5500 mile pipeline system that delivers 2.5 million barrels of fuel per day and critical liquid fuel infrastructure from oil refineries to states along the U.S. East Coast (Brower and McCormick 2021). These and other cyber incidents have led the U.S. to strengthen its cybersecurity and introduce, among other things, a public body to analyse major cyber incidents and make recommendations to prevent a recurrence (Murphey 2021a). Another example of the scope of cyberattacks is the ransomware NotPetya in 2017. The damage amounted to USD 10 billion, as the ransomware exploited a vulnerability in the windows system, allowing it to spread independently worldwide in the network (GAO 2021). In the same year, the ransomware WannaCry was launched by cybercriminals. The cyberattack on Windows software took user data hostage in exchange for Bitcoin cryptocurrency (Smart 2018). The victims included the National Health Service in Great Britain. As a result, ambulances were redirected to other hospitals because of information technology (IT) systems failing, leaving people in need of urgent assistance waiting. It has been estimated that 19,000 cancelled treatment appointments resulted from losses of GBP 92 million (Field 2018). Throughout the COVID-19 pandemic, ransomware attacks increased significantly, as working from home arrangements increased vulnerability (Murphey 2021b).

Besides cyberattacks, data breaches can also cause high costs. Under the General Data Protection Regulation (GDPR), companies are obliged to protect personal data and safeguard the data protection rights of all individuals in the EU area. The GDPR allows data protection authorities in each country to impose sanctions and fines on organisations they find in breach. “For data breaches, the maximum fine can be €20 million or 4% of global turnover, whichever is higher” (GDPR.EU 2021). Data breaches often involve a large amount of sensitive data that has been accessed, unauthorised, by external parties, and are therefore considered important for information security due to their far-reaching impact (Goode et al. 2017). A data breach is defined as a “security incident in which sensitive, protected, or confidential data are copied, transmitted, viewed, stolen, or used by an unauthorized individual” (Freeha et al. 2021). Depending on the amount of data, the extent of the damage caused by a data breach can be significant, with the average cost being USD 392 million^1^ (IBM Security 2020).

This research paper reviews the existing literature and open data sources related to cybersecurity and cyber risk, focusing on the datasets used to improve academic understanding and advance the current state-of-the-art in cybersecurity. Furthermore, important information about the available datasets is presented (e.g. use cases), and a plea is made for open data and the standardisation of cyber risk data for academic comparability and replication. The remainder of the paper is structured as follows. The next section describes the related work regarding cybersecurity and cyber risks. The third section outlines the review method used in this work and the process. The fourth section details the results of the identified literature. Further discussion is presented in the penultimate section and the final section concludes.

## Related work

Due to the significance of cyber risks, several literature reviews have been conducted in this field. Eling (2020) reviewed the existing academic literature on the topic of cyber risk and cyber insurance from an economic perspective. A total of 217 papers with the term ‘cyber risk’ were identified and classified in different categories. As a result, open research questions are identified, showing that research on cyber risks is still in its infancy because of their dynamic and emerging nature. Furthermore, the author highlights that particular focus should be placed on the exchange of information between public and private actors. An improved information flow could help to measure the risk more accurately and thus make cyber risks more insurable and help risk managers to determine the right level of cyber risk for their company. In the context of cyber insurance data, Romanosky et al. (2019) analysed the underwriting process for cyber insurance and revealed how cyber insurers understand and assess cyber risks. For this research, they examined 235 American cyber insurance policies that were publicly available and looked at three components (coverage, application questionnaires and pricing). The authors state in their findings that many of the insurers used very simple, flat-rate pricing (based on a single calculation of expected loss), while others used more parameters such as the asset value of the company (or company revenue) or standard insurance metrics (e.g. deductible, limits), and the industry in the calculation. This is in keeping with Eling (2020), who states that an increased amount of data could help to make cyber risk more accurately measured and thus more insurable. Similar research on cyber insurance and data was conducted by Nurse et al. (2020). The authors examined cyber insurance practitioners' perceptions and the challenges they face in collecting and using data. In addition, gaps were identified during the research where further data is needed. The authors concluded that cyber insurance is still in its infancy, and there are still several unanswered questions (for example, cyber valuation, risk calculation and recovery). They also pointed out that a better understanding of data collection and use in cyber insurance would be invaluable for future research and practice. Bessy-Roland et al. (2021) come to a similar conclusion. They proposed a multivariate Hawkes framework to model and predict the frequency of cyberattacks. They used a public dataset with characteristics of data breaches affecting the U.S. industry. In the conclusion, the authors make the argument that an insurer has a better knowledge of cyber losses, but that it is based on a small dataset and therefore combination with external data sources seems essential to improve the assessment of cyber risks.

Several systematic reviews have been published in the area of cybersecurity (Kruse et al. 2017; Lee et al. 2020; Loukas et al. 2013; Ulven and Wangen 2021). In these papers, the authors concentrated on a specific area or sector in the context of cybersecurity. This paper adds to this extant literature by focusing on data availability and its importance to risk management and insurance stakeholders. With a priority on healthcare and cybersecurity, Kruse et al. (2017) conducted a systematic literature review. The authors identified 472 articles with the keywords ‘cybersecurity and healthcare’ or ‘ransomware’ in the databases Cumulative Index of Nursing and Allied Health Literature, PubMed and Proquest. Articles were eligible for this review if they satisfied three criteria: (1) they were published between 2006 and 2016, (2) the full-text version of the article was available, and (3) the publication is a peer-reviewed or scholarly journal. The authors found that technological development and federal policies (in the U.S.) are the main factors exposing the health sector to cyber risks. Loukas et al. (2013) conducted a review with a focus on cyber risks and cybersecurity in emergency management. The authors provided an overview of cyber risks in communication, sensor, information management and vehicle technologies used in emergency management and showed areas for which there is still no solution in the literature. Similarly, Ulven and Wangen (2021) reviewed the literature on cybersecurity risks in higher education institutions. For the literature review, the authors used the keywords ‘cyber’, ‘information threats’ or ‘vulnerability’ in connection with the terms ‘higher education, ‘university’ or ‘academia’. A similar literature review with a focus on Internet of Things (IoT) cybersecurity was conducted by Lee et al. (2020). The review revealed that qualitative approaches focus on high-level frameworks, and quantitative approaches to cybersecurity risk management focus on risk assessment and quantification of cyberattacks and impacts. In addition, the findings presented a four-step IoT cyber risk management framework that identifies, quantifies and prioritises cyber risks.

Datasets are an essential part of cybersecurity research, underlined by the following works. Ilhan Firat et al. (2021) examined various cybersecurity datasets in detail. The study was motivated by the fact that with the proliferation of the internet and smart technologies, the mode of cyberattacks is also evolving. However, in order to prevent such attacks, they must first be detected; the dissemination and further development of cybersecurity datasets is therefore critical. In their work, the authors observed studies of datasets used in intrusion detection systems. Khraisat et al. (2019) also identified a need for new datasets in the context of cybersecurity. The researchers presented a taxonomy of current intrusion detection systems, a comprehensive review of notable recent work, and an overview of the datasets commonly used for assessment purposes. In their conclusion, the authors noted that new datasets are needed because most machine-learning techniques are trained and evaluated on the knowledge of old datasets. These datasets do not contain new and comprehensive information and are partly derived from datasets from 1999. The authors noted that the core of this issue is the availability of new public datasets as well as their quality. The availability of data, how it is used, created and shared was also investigated by Zheng et al. (2018). The researchers analysed 965 cybersecurity research papers published between 2012 and 2016. They created a taxonomy of the types of data that are created and shared and then analysed the data collected via datasets. The researchers concluded that while datasets are recognised as valuable for cybersecurity research, the proportion of publicly available datasets is limited.

The main contributions of this review and what differentiates it from previous studies can be summarised as follows. First, as far as we can tell, it is the first work to summarise all available datasets on cyber risk and cybersecurity in the context of a systematic review and present them to the scientific community and cyber insurance and cybersecurity stakeholders. Second, we investigated, analysed, and made available the datasets to support efficient and timely progress in cyber risk research. And third, we enable comparability of datasets so that the appropriate dataset can be selected depending on the research area.

## Methodology

### Process and eligibility criteria

The structure of this systematic review is inspired by the Preferred Reporting Items for Systematic Reviews and Meta-Analyses (PRISMA) framework (Page et al. 2021), and the search was conducted from 3 to 10 May 2021. Due to the continuous development of cyber risks and their countermeasures, only articles published in the last 10 years were considered. In addition, only articles published in peer-reviewed journals written in English were included. As a final criterion, only articles that make use of one or more cybersecurity or cyber risk datasets met the inclusion criteria. Specifically, these studies presented new or existing datasets, used them for methods, or used them to verify new results, as well as analysed them in an economic context and pointed out their effects. The criterion was fulfilled if it was clearly stated in the abstract that one or more datasets were used. A detailed explanation of this selection criterion can be found in the ‘Study selection’ section.

### Information sources

In order to cover a complete spectrum of literature, various databases were queried to collect relevant literature on the topic of cybersecurity and cyber risks. Due to the spread of related articles across multiple databases, the literature search was limited to the following four databases for simplicity: IEEE Xplore, Scopus, SpringerLink and Web of Science. This is similar to other literature reviews addressing cyber risks or cybersecurity, including Sardi et al. (2021), Franke and Brynielsson (2014), Lagerström (2019), Eling and Schnell (2016) and Eling (2020). In this paper, all databases used in the aforementioned works were considered. However, only two studies also used all the databases listed. The IEEE Xplore database contains electrical engineering, computer science, and electronics work from over 200 journals and three million conference papers (IEEE 2021). Scopus includes 23,400 peer-reviewed journals from more than 5000 international publishers in the areas of science, engineering, medicine, social sciences and humanities (Scopus 2021). SpringerLink contains 3742 journals and indexes over 10 million scientific documents (SpringerLink 2021). Finally, Web of Science indexes over 9200 journals in different scientific disciplines (Science 2021).

### Search

A search string was created and applied to all databases. To make the search efficient and reproducible, the following search string with Boolean operator was used in all databases: cybersecurity OR cyber risk AND dataset OR database. To ensure uniformity of the search across all databases, some adjustments had to be made for the respective search engines. In Scopus, for example, the Advanced Search was used, and the field code ‘Title-ABS-KEY’ was integrated into the search string. For IEEE Xplore, the search was carried out with the Search String in the Command Search and ‘All Metadata’. In the Web of Science database, the Advanced Search was used. The special feature of this search was that it had to be carried out in individual steps. The first search was carried out with the terms cybersecurity OR cyber risk with the field tag Topic (T.S. =) and the second search with dataset OR database. Subsequently, these searches were combined, which then delivered the searched articles for review. For SpringerLink, the search string was used in the Advanced Search under the category ‘Find the resources with all of the words’. After conducting this search string, 5219 studies could be found. According to the eligibility criteria (period, language and only scientific journals), 1581 studies were identified in the databases:

- IEEE: 364
- Scopus: 135
- Springer Link: 548
- Web of Science: 534

**Fig. 2:**
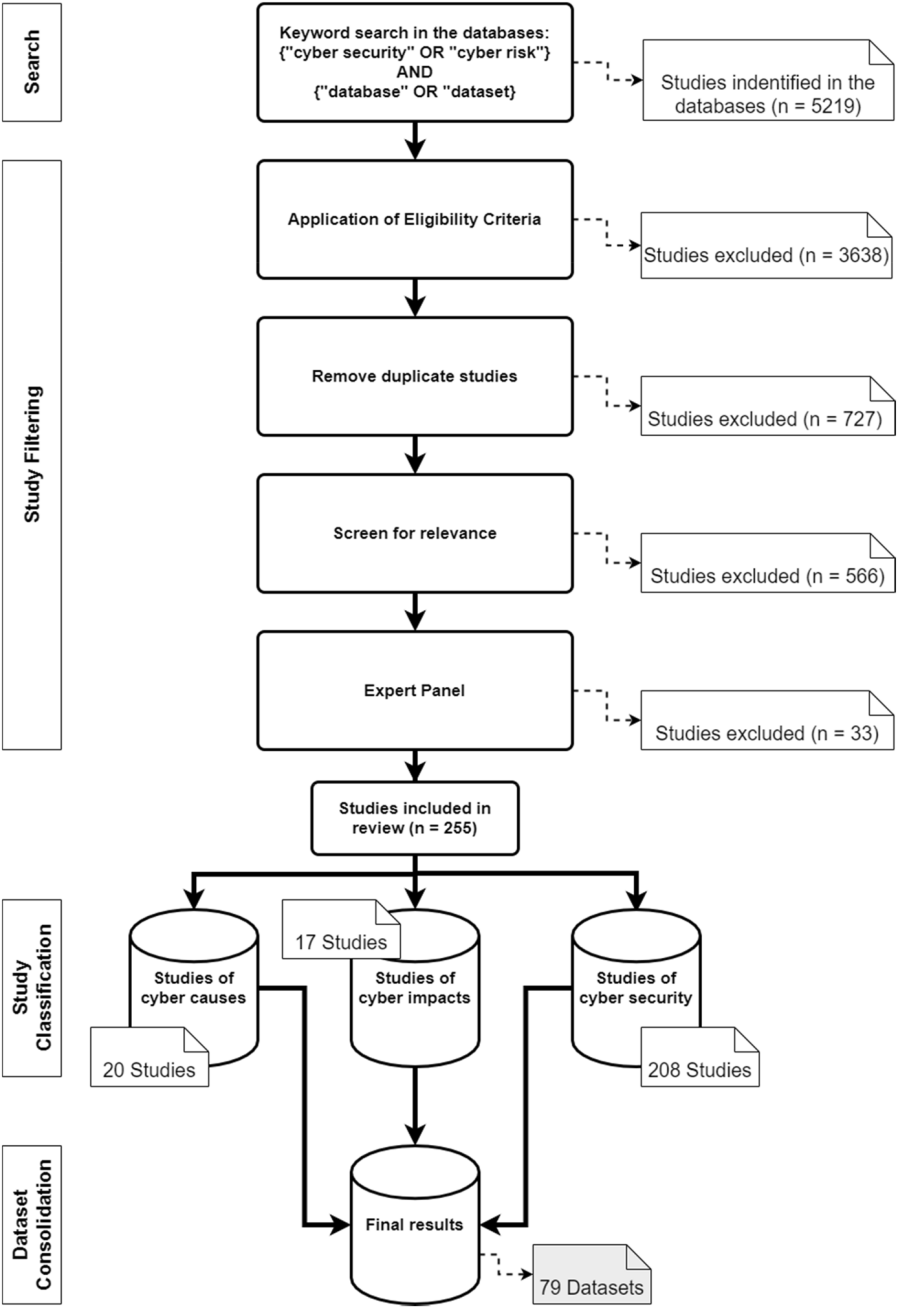
Literature search process and categorisation of the studies

An overview of the process is given in Fig. 2. Combined with the results from the four databases, 854 articles without duplicates were identified.

### Study selection

**Fig. 3:**
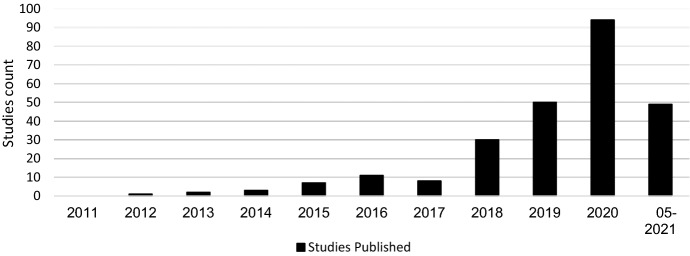
Distribution of studies

In the final step of the selection process, the articles were screened for relevance. Due to a large number of results, the abstracts were analysed in the first step of the process. The aim was to determine whether the article was relevant for the systematic review. An article fulfilled the criterion if it was recognisable in the abstract that it had made a contribution to datasets or databases with regard to cyber risks or cybersecurity. Specifically, the criterion was considered to be met if the abstract used datasets that address the causes or impacts of cyber risks, and measures in the area of cybersecurity. In this process, the number of articles was reduced to 288. The articles were then read in their entirety, and an expert panel of six people decided whether they should be used. This led to a final number of 255 articles. The years in which the articles were published and the exact number can be seen in Fig. 3.

### Data collection process and synthesis of the results

For the data collection process, various data were extracted from the studies, including the names of the respective creators, the name of the dataset or database and the corresponding reference. It was also determined where the data came from. In the context of accessibility, it was determined whether access is free, controlled, available for purchase or not available. It was also determined when the datasets were created and the time period referenced. The application type and domain characteristics of the datasets were identified.

## Results

**Fig. 4:**
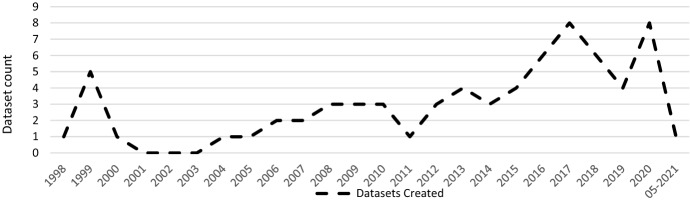
Distribution of dataset results

**Fig. 5:**
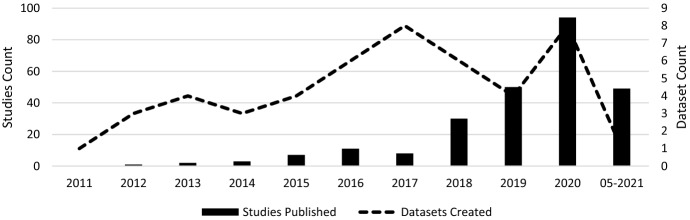
Correlation between the studies and the datasets

**Fig. 6:**
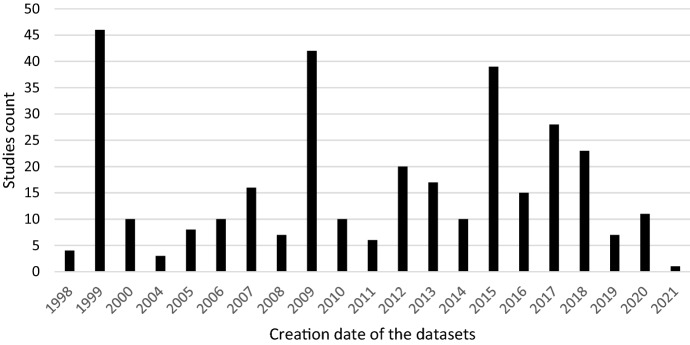
Distribution of studies and their use of datasets

This section analyses the results of the systematic literature review. The previously identified studies are divided into three categories: datasets on the causes of cyber risks, datasets on the effects of cyber risks and datasets on cybersecurity. The classification is based on the intended use of the studies. This system of classification makes it easier for stakeholders to find the appropriate datasets. The categories are evaluated individually. Although complete information is available for a large proportion of datasets, this is not true for all of them. Accordingly, the abbreviation N/A has been inserted in the respective characters to indicate that this information could not be determined by the time of submission. The term ‘use cases in the literature’ in the following and supplementary tables refers to the application areas in which the corresponding datasets were used in the literature. The areas listed there refer to the topic area on which the researchers conducted their research. Since some datasets were used interdisciplinarily, the listed use cases in the literature are correspondingly longer. Before discussing each category in the next sections, Fig. 4 provides an overview of the number of datasets found and their year of creation. Figure 5 then shows the relationship between studies and datasets in the period under consideration. Figure 6 shows the distribution of studies, their use of datasets and their creation date. The number of datasets used is higher than the number of studies because the studies often used several datasets (Table 1).

**Table 1:** Percentage contribution of datasets for each place of origin

Rank	Place of origin	Percentage of datasets
1	U.S.	58.2
2	Canada	11.3
3	Australia	5
4	Germany	3.7
5	U.K.	3.7
6	France	2.5
7	Italy	2.5
8	Spain	2.5
9	China	1.2
10	Czech Republic	1.2
11	Greece	1.2
12	Japan	1.2
13	Lithuania	1.2
14	Luxembourg	1.2
15	Netherlands	1.2
16	Republic of Korea	1.2
17	Turkey	1.2

Most of the datasets are generated in the U.S. (up to 58.2%). Canada and Australia rank next, with 11.3% and 5% of all the reviewed datasets, respectively.

Additionally, to create value for the datasets for the cyber insurance industry, an assessment of the applicability of each dataset has been provided for cyber insurers. This ‘Use Case Assessment’ includes the use of the data in the context of different analyses, calculation of cyber insurance premiums, and use of the information for the design of cyber insurance contracts or for additional customer services. To reasonably account for the transition of direct hyperlinks in the future, references were directed to the main websites for longevity (nearest resource point). In addition, the links to the main pages contain further information on the datasets and different versions related to the operating systems. The references were chosen in such a way that practitioners get the best overview of the respective datasets.

### Case datasets

This section presents selected articles that use the datasets to analyse the causes of cyber risks. The datasets help identify emerging trends and allow pattern discovery in cyber risks. This information gives cybersecurity experts and cyber insurers the data to make better predictions and take appropriate action. For example, if certain vulnerabilities are not adequately protected, cyber insurers will demand a risk surcharge leading to an improvement in the risk-adjusted premium. Due to the capricious nature of cyber risks, existing data must be supplemented with new data sources (for example, new events, new methods or security vulnerabilities) to determine prevailing cyber exposure. The datasets of cyber risk causes could be combined with existing portfolio data from cyber insurers and integrated into existing pricing tools and factors to improve the valuation of cyber risks.

A portion of these datasets consists of several taxonomies and classifications of cyber risks. Aassal et al. (2020) propose a new taxonomy of phishing characteristics based on the interpretation and purpose of each characteristic. In comparison, Hindy et al. (2020) presented a taxonomy of network threats and the impact of current datasets on intrusion detection systems. A similar taxonomy was suggested by Kiwia et al. (2018). The authors presented a cyber kill chain-based taxonomy of banking Trojans features. The taxonomy built on a real-world dataset of 127 banking Trojans collected from December 2014 to January 2016 by a major U.K.-based financial organisation.

In the context of classification, Aamir et al. (2021) showed the benefits of machine learning for classifying port scans and DDoS attacks in a mixture of normal and attack traffic. Guo et al. (2020) presented a new method to improve malware classification based on entropy sequence features. The evaluation of this new method was conducted on different malware datasets.

To reconstruct attack scenarios and draw conclusions based on the evidence in the alert stream, Barzegar and Shajari (2018) use the DARPA2000 and MACCDC 2012 dataset for their research. Giudici and Raffinetti (2020) proposed a rank-based statistical model aimed at predicting the severity levels of cyber risk. The model used cyber risk data from the University of Milan. In contrast to the previous datasets, Skrjanc et al. (2018) used the older dataset KDD99 to monitor large-scale cyberattacks using a cauchy clustering method.

Amin et al. (2021) used a cyberattack dataset from the Canadian Institute for Cybersecurity to identify spatial clusters of countries with high rates of cyberattacks. In the context of cybercrime, Junger et al. (2020) examined crime scripts, key characteristics of the target company and the relationship between criminal effort and financial benefit. For their study, the authors analysed 300 cases of fraudulent activities against Dutch companies. With a similar focus on cybercrime, Mireles et al. (2019) proposed a metric framework to measure the effectiveness of the dynamic evolution of cyberattacks and defensive measures. To validate its usefulness, they used the DEFCON dataset.

Due to the rapidly changing nature of cyber risks, it is often impossible to obtain all information on them. Kim and Kim (2019) proposed an automated dataset generation system called CTIMiner that collects threat data from publicly available security reports and malware repositories. They released a dataset to the public containing about 640,000 records from 612 security reports published between January 2008 and 2019. A similar approach is proposed by Kim et al. (2020), using a named entity recognition system to extract core information from cyber threat reports automatically. They created a 498,000-tag dataset during their research (Ulven and Wangen 2021).

Within the framework of vulnerabilities and cybersecurity issues, Ulven and Wangen (2021) proposed an overview of mission-critical assets and everyday threat events, suggested a generic threat model, and summarised common cybersecurity vulnerabilities. With a focus on hospitality, Chen and Fiscus (2018) proposed several issues related to cybersecurity in this sector. They analysed 76 security incidents from the Privacy Rights Clearinghouse database. Supplementary Table 1 lists all findings that belong to the cyber causes dataset.

### Impact datasets

This section outlines selected findings of the cyber impact dataset. For cyber insurers, these datasets can form an important basis for information, as they can be used to calculate cyber insurance premiums, evaluate specific cyber risks, formulate inclusions and exclusions in cyber wordings, and re-evaluate as well as supplement the data collected so far on cyber risks. For example, information on financial losses can help to better assess the loss potential of cyber risks. Furthermore, the datasets can provide insight into the frequency of occurrence of these cyber risks. The new datasets can be used to close any data gaps that were previously based on very approximate estimates or to find new results.

Eight studies addressed the costs of data breaches. For instance, Eling and Jung (2018) reviewed 3327 data breach events from 2005 to 2016 and identified an asymmetric dependence of monthly losses by breach type and industry. The authors used datasets from the Privacy Rights Clearinghouse for analysis. The Privacy Rights Clearinghouse datasets and the Breach level index database were also used by De Giovanni et al. (2020) to describe relationships between data breaches and bitcoin-related variables using the cointegration methodology. The data were obtained from the Department of Health and Human Services of healthcare facilities reporting data breaches and a national database of technical and organisational infrastructure information. Also in the context of data breaches, Algarni et al. (2021) developed a comprehensive, formal model that estimates the two components of security risks: breach cost and the likelihood of a data breach within 12 months. For their survey, the authors used two industrial reports from the Ponemon institute and VERIZON. To illustrate the scope of data breaches, Neto et al. (2021) identified 430 major data breach incidents among more than 10,000 incidents. The database created is available and covers the period 2018 to 2019.

With a direct focus on insurance, Biener et al. (2015) analysed 994 cyber loss cases from an operational risk database and investigated the insurability of cyber risks based on predefined criteria. For their study, they used data from the company SAS OpRisk Global Data. Similarly, Eling and Wirfs (2019) looked at a wide range of cyber risk events and actual cost data using the same database. They identified cyber losses and analysed them using methods from statistics and actuarial science. Using a similar reference, Farkas et al. (2021) proposed a method for analysing cyber claims based on regression trees to identify criteria for classifying and evaluating claims. Similar to Chen and Fiscus (2018), the dataset used was the Privacy Rights Clearinghouse database. Within the framework of reinsurance, Moro (2020) analysed cyber index-based information technology activity to see if index-parametric reinsurance coverage could suggest its cedant using data from a Symantec dataset.

Paté-Cornell et al. (2018) presented a general probabilistic risk analysis framework for cybersecurity in an organisation to be specified. The results are distributions of losses to cyberattacks, with and without considered countermeasures in support of risk management decisions based both on past data and anticipated incidents. The data used were from The Common Vulnerability and Exposures database and via confidential access to a database of cyberattacks on a large, U.S.-based organisation. A different conceptual framework for cyber risk classification and assessment was proposed by Sheehan et al. (2021). This framework showed the importance of proactive and reactive barriers in reducing companies’ exposure to cyber risk and quantifying the risk. Another approach to cyber risk assessment and mitigation was proposed by Mukhopadhyay et al. (2019). They estimated the probability of an attack using generalised linear models, predicted the security technology required to reduce the probability of cyberattacks, and used gamma and exponential distributions to best approximate the average loss data for each malicious attack. They also calculated the expected loss due to cyberattacks, calculated the net premium that would need to be charged by a cyber insurer, and suggested cyber insurance as a strategy to minimise losses. They used the CSI-FBI survey (1997–2010) to conduct their research.

In order to highlight the lack of data on cyber risks, Eling (2020) conducted a literature review in the areas of cyber risk and cyber insurance. Available information on the frequency, severity, and dependency structure of cyber risks was filtered out. In addition, open questions for future cyber risk research were set up. Another example of data collection on the impact of cyberattacks is provided by Sornette et al. (2013), who use a database of newspaper articles, press reports and other media to provide a predictive method to identify triggering events and potential accident scenarios and estimate their severity and frequency. A similar approach to data collection was used by Arcuri et al. (2020) to gather an original sample of global cyberattacks from newspaper reports sourced from the LexisNexis database. This collection is also used and applied to the fields of dynamic communication and cyber risk perception by Fang et al. (2021). To create a dataset of cyber incidents and disputes, Valeriano and Maness (2014) collected information on cyber interactions between rival states.

To assess trends and the scale of economic cybercrime, Levi (2017) examined datasets from different countries and their impact on crime policy. Pooser et al. (2018) investigated the trend in cyber risk identification from 2006 to 2015 and company characteristics related to cyber risk perception. The authors used a dataset of various reports from cyber insurers for their study. Walker-Roberts et al. (2020) investigated the spectrum of risk of a cybersecurity incident taking place in the cyber-physical-enabled world using the VERIS Community Database. The datasets of impacts identified are presented below. Due to overlap, some may also appear in the causes dataset (Supplementary Table 2).

### Cybersecurity datasets

#### General intrusion detection

General intrusion detection systems account for the largest share of countermeasure datasets. For companies or researchers focused on cybersecurity, the datasets can be used to test their own countermeasures or obtain information about potential vulnerabilities. For example, Al-Omari et al. (2021) proposed an intelligent intrusion detection model for predicting and detecting attacks in cyberspace, which was applied to dataset UNSW-NB 15. A similar approach was taken by Choras and Kozik (2015), who used machine learning to detect cyberattacks on web applications. To evaluate their method, they used the HTTP dataset CSIC 2010. For the identification of unknown attacks on web servers, Kamarudin et al. (2017) proposed an anomaly-based intrusion detection system using an ensemble classification approach. Ganeshan and Rodrigues (2020) showed an intrusion detection system approach, which clusters the database into several groups and detects the presence of intrusion in the clusters. In comparison, AlKadi et al. (2019) used a localisation-based model to discover abnormal patterns in network traffic. Hybrid models have been recommended by Bhattacharya et al. (2020) and Agrawal et al. (2019); the former is a machine-learning model based on principal component analysis for the classification of intrusion detection system datasets, while the latter is a hybrid ensemble intrusion detection system for anomaly detection using different datasets to detect patterns in network traffic that deviate from normal behaviour.

Agarwal et al. (2021) used three different machine learning algorithms in their research to find the most suitable for efficiently identifying patterns of suspicious network activity. The UNSW-NB15 dataset was used for this purpose. Kasongo and Sun (2020), Feed-Forward Deep Neural Network (FFDNN), Keshk et al. (2021), the privacy-preserving anomaly detection framework, and others also use the UNSW-NB 15 dataset as part of intrusion detection systems. The same dataset and others were used by Binbusayyis and Vaiyapuri (2019) to identify and compare key features for cyber intrusion detection. Atefinia and Ahmadi (2021) proposed a deep neural network model to reduce the false positive rate of an anomaly-based intrusion detection system. Fossaceca et al. (2015) focused in their research on the development of a framework that combined the outputs of multiple learners in order to improve the efficacy of network intrusion, and Gauthama Raman et al. (2020) presented a search algorithm based on Support Vector machine to improve the performance of the detection and false alarm rate to improve intrusion detection techniques. Ahmad and Alsemmeari (2020) targeted extreme learning machine techniques due to their good capabilities in classification problems and handling huge data. They used the NSL-KDD dataset as a benchmark.

With reference to prediction, Bakdash et al. (2018) used datasets from the U.S. Department of Defence to predict cyberattacks by malware. This dataset consists of weekly counts of cyber events over approximately seven years. Another prediction method was presented by Fan et al. (2018), which showed an improved integrated cybersecurity prediction method based on spatial-time analysis. Also, with reference to prediction, Ashtiani and Azgomi (2014) proposed a framework for the distributed simulation of cyberattacks based on high-level architecture. Kirubavathi and Anitha (2016) recommended an approach to detect botnets, irrespective of their structures, based on network traffic flow behaviour analysis and machine-learning techniques. Dwivedi et al. (2021) introduced a multi-parallel adaptive technique to utilise an adaption mechanism in the group of swarms for network intrusion detection. AlEroud and Karabatis (2018) presented an approach that used contextual information to automatically identify and query possible semantic links between different types of suspicious activities extracted from network flows.

#### Intrusion detection systems with a focus on IoT

In addition to general intrusion detection systems, a proportion of studies focused on IoT. Habib et al. (2020) presented an approach for converting traditional intrusion detection systems into smart intrusion detection systems for IoT networks. To enhance the process of diagnostic detection of possible vulnerabilities with an IoT system, Georgescu et al. (2019) introduced a method that uses a named entity recognition-based solution. With regard to IoT in the smart home sector, Heartfield et al. (2021) presented a detection system that is able to autonomously adjust the decision function of its underlying anomaly classification models to a smart home’s changing condition. Another intrusion detection system was suggested by Keserwani et al. (2021), which combined Grey Wolf Optimization and Particle Swam Optimization to identify various attacks for IoT networks. They used the KDD Cup 99, NSL-KDD and CICIDS-2017 to evaluate their model. Abu Al-Haija and Zein-Sabatto (2020) provide a comprehensive development of a new intelligent and autonomous deep-learning-based detection and classification system for cyberattacks in IoT communication networks that leverage the power of convolutional neural networks, abbreviated as IoT-IDCS-CNN (IoT-based Intrusion Detection and Classification System using Convolutional Neural Network). To evaluate the development, the authors used the NSL-KDD dataset. Biswas and Roy (2021) recommended a model that identifies malicious botnet traffic using novel deep-learning approaches like artificial neural networks gutted recurrent units and long- or short-term memory models. They tested their model with the Bot-IoT dataset.

With a more forensic background, Koroniotis et al. (2020) submitted a network forensic framework, which described the digital investigation phases for identifying and tracing attack behaviours in IoT networks. The suggested work was evaluated with the Bot-IoT and UINSW-NB15 datasets. With a focus on big data and IoT, Chhabra et al. (2020) presented a cyber forensic framework for big data analytics in an IoT environment using machine learning. Furthermore, the authors mentioned different publicly available datasets for machine-learning models.

A stronger focus on a mobile phones was exhibited by Alazab et al. (2020), which presented a classification model that combined permission requests and application programme interface calls. The model was tested with a malware dataset containing 27,891 Android apps. A similar approach was taken by Li et al. (2019a, b), who proposed a reliable classifier for Android malware detection based on factorisation machine architecture and extraction of Android app features from manifest files and source code.

#### Literature reviews

In addition to the different methods and models for intrusion detection systems, various literature reviews on the methods and datasets were also found. Liu and Lang (2019) proposed a taxonomy of intrusion detection systems that uses data objects as the main dimension to classify and summarise machine learning and deep learning-based intrusion detection literature. They also presented four different benchmark datasets for machine-learning detection systems. Ahmed et al. (2016) presented an in-depth analysis of four major categories of anomaly detection techniques, which include classification, statistical, information theory and clustering. Hajj et al. (2021) gave a comprehensive overview of anomaly-based intrusion detection systems. Their article gives an overview of the requirements, methods, measurements and datasets that are used in an intrusion detection system.

Within the framework of machine learning, Chattopadhyay et al. (2018) conducted a comprehensive review and meta-analysis on the application of machine-learning techniques in intrusion detection systems. They also compared different machine learning techniques in different datasets and summarised the performance. Vidros et al. (2017) presented an overview of characteristics and methods in automatic detection of online recruitment fraud. They also published an available dataset of 17,880 annotated job ads, retrieved from the use of a real-life system. An empirical study of different unsupervised learning algorithms used in the detection of unknown attacks was presented by Meira et al. (2020).

#### New datasets

Kilincer et al. (2021) reviewed different intrusion detection system datasets in detail. They had a closer look at the UNS-NB15, ISCX-2012, NSL-KDD and CIDDS-001 datasets. Stojanovic et al. (2020) also provided a review on datasets and their creation for use in advanced persistent threat detection in the literature. Another review of datasets was provided by Sarker et al. (2020), who focused on cybersecurity data science as part of their research and provided an overview from a machine-learning perspective. Avila et al. (2021) conducted a systematic literature review on the use of security logs for data leak detection. They recommended a new classification of information leak, which uses the GDPR principles, identified the most widely publicly available dataset for threat detection, described the attack types in the datasets and the algorithms used for data leak detection. Tuncer et al. (2020) presented a bytecode-based detection method consisting of feature extraction using local neighbourhood binary patterns. They chose a byte-based malware dataset to investigate the performance of the proposed local neighbourhood binary pattern-based detection method. With a different focus, Mauro et al. (2020) gave an experimental overview of neural-based techniques relevant to intrusion detection. They assessed the value of neural networks using the Bot-IoT and UNSW-DB15 datasets.

Another category of results in the context of countermeasure datasets is those that were presented as new. Moreno et al. (2018) developed a database of 300 security-related accidents from European and American sources. The database contained cybersecurity-related events in the chemical and process industry. Damasevicius et al. (2020) proposed a new dataset (LITNET-2020) for network intrusion detection. The dataset is a new annotated network benchmark dataset obtained from the real-world academic network. It presents real-world examples of normal and under-attack network traffic. With a focus on IoT intrusion detection systems, Alsaedi et al. (2020) proposed a new benchmark IoT/IIot datasets for assessing intrusion detection system-enabled IoT systems. Also in the context of IoT, Vaccari et al. (2020) proposed a dataset focusing on message queue telemetry transport protocols, which can be used to train machine-learning models. To evaluate the performance of machine-learning classifiers, Mahfouz et al. (2020) created a dataset called Game Theory and Cybersecurity (GTCS). A dataset containing 22,000 malware and benign samples was constructed by Martin et al. (2019). The dataset can be used as a benchmark to test the algorithm for Android malware classification and clustering techniques. In addition, Laso et al. (2017) presented a dataset created to investigate how data and information quality estimates enable the detection of anomalies and malicious acts in cyber-physical systems. The dataset contained various cyberattacks and is publicly available.

#### Other

In addition to the results described above, several other studies were found that fit into the category of countermeasures. Johnson et al. (2016) examined the time between vulnerability disclosures. Using another vulnerabilities database, Common Vulnerabilities and Exposures (CVE), Subroto and Apriyana (2019) presented an algorithm model that uses big data analysis of social media and statistical machine learning to predict cyber risks. A similar databank but with a different focus, Common Vulnerability Scoring System, was used by Chatterjee and Thekdi (2020) to present an iterative data-driven learning approach to vulnerability assessment and management for complex systems. Using the CICIDS2017 dataset to evaluate the performance, Malik et al. (2020) proposed a control plane-based orchestration for varied, sophisticated threats and attacks. The same dataset was used in another study by Lee et al. (2019), who developed an artificial security information event management system based on a combination of event profiling for data processing and different artificial network methods. To exploit the interdependence between multiple series, Fang et al. (2021) proposed a statistical framework. In order to validate the framework, the authors applied it to a dataset of enterprise-level security breaches from the Privacy Rights Clearinghouse and Identity Theft Center database. Another framework with a defensive aspect was recommended by Li et al. (2021) to increase the robustness of deep neural networks against adversarial malware evasion attacks. Sarabi et al. (2016) investigated whether and to what extent business details can help assess an organisation's risk of data breaches and the distribution of risk across different types of incidents to create policies for protection, detection and recovery from different forms of security incidents. They used data from the VERIS Community Database.

Datasets that have been classified into the cybersecurity category are detailed in Supplementary Table 3. Due to overlap, records from the previous tables may also be included.

## Discussion

This paper presented a systematic literature review of studies on cyber risk and cybersecurity that used datasets. Within this framework, 255 studies were fully reviewed and then classified into three different categories. Then, 79 datasets were consolidated from these studies. These datasets were subsequently analysed, and important information was selected through a process of filtering out. This information was recorded in a table and enhanced with further information as part of the literature analysis. This made it possible to create a comprehensive overview of the datasets. For example, each dataset contains a description of where the data came from and how the data has been used to date. This allows different datasets to be compared and the appropriate dataset for the use case to be selected. This research certainly has limitations, so our selection of datasets cannot necessarily be taken as a representation of all available datasets related to cyber risks and cybersecurity. For example, literature searches were conducted in four academic databases and only found datasets that were used in the literature. Many research projects also used old datasets that may no longer consider current developments. In addition, the data are often focused on only one observation and are limited in scope. For example, the datasets can only be applied to specific contexts and are also subject to further limitations (e.g. region, industry, operating system). In the context of the applicability of the datasets, it is unfortunately not possible to make a clear statement on the extent to which they can be integrated into academic or practical areas of application or how great this effort is. Finally, it remains to be pointed out that this is an overview of currently available datasets, which are subject to constant change.

Due to the lack of datasets on cyber risks in the academic literature, additional datasets on cyber risks were integrated as part of a further search. The search was conducted on the Google Dataset search portal. The search term used was ‘cyber risk datasets’. Over 100 results were found. However, due to the low significance and verifiability, only 20 selected datasets were included. These can be found in Table 2 in the “Appendix”.

The results of the literature review and datasets also showed that there continues to be a lack of available, open cyber datasets. This lack of data is reflected in cyber insurance, for example, as it is difficult to find a risk-based premium without a sufficient database (Nurse et al. 2020). The global cyber insurance market was estimated at USD 5.5 billion in 2020 (Dyson 2020). When compared to the USD 1 trillion global losses from cybercrime (Maleks Smith et al. 2020), it is clear that there exists a significant cyber risk awareness challenge for both the insurance industry and international commerce. Without comprehensive and qualitative data on cyber losses, it can be difficult to estimate potential losses from cyberattacks and price cyber insurance accordingly (GAO 2021). For instance, the average cyber insurance loss increased from USD 145,000 in 2019 to USD 359,000 in 2020 (FitchRatings 2021). Cyber insurance is an important risk management tool to mitigate the financial impact of cybercrime. This is particularly evident in the impact of different industries. In the Energy & Commodities financial markets, a ransomware attack on the Colonial Pipeline led to a substantial impact on the U.S. economy. As a result of the attack, about 45% of the U.S. East Coast was temporarily unable to obtain supplies of diesel, petrol and jet fuel. This caused the average price in the U.S. to rise 7 cents to USD 3.04 per gallon, the highest in seven years (Garber 2021). In addition, Colonial Pipeline confirmed that it paid a USD 4.4 million ransom to a hacker gang after the attack. Another ransomware attack occurred in the healthcare and government sector. The victim of this attack was the Irish Health Service Executive (HSE). A ransom payment of USD 20 million was demanded from the Irish government to restore services after the hack (Tidy 2021). In the car manufacturing sector, Miller and Valasek (2015) initiated a cyberattack that resulted in the recall of 1.4 million vehicles and cost manufacturers EUR 761 million. The risk that arises in the context of these events is the potential for the accumulation of cyber losses, which is why cyber insurers are not expanding their capacity. An example of this accumulation of cyber risks is the NotPetya malware attack, which originated in Russia, struck in Ukraine, and rapidly spread around the world, causing at least USD 10 billion in damage (GAO 2021). These events highlight the importance of proper cyber risk management.

This research provides cyber insurance stakeholders with an overview of cyber datasets. Cyber insurers can use the open datasets to improve their understanding and assessment of cyber risks. For example, the impact datasets can be used to better measure financial impacts and their frequencies. These data could be combined with existing portfolio data from cyber insurers and integrated with existing pricing tools and factors to better assess cyber risk valuation. Although most cyber insurers have sparse historical cyber policy and claims data, they remain too small at present for accurate prediction (Bessy-Roland et al. 2021). A combination of portfolio data and external datasets would support risk-adjusted pricing for cyber insurance, which would also benefit policyholders. In addition, cyber insurance stakeholders can use the datasets to identify patterns and make better predictions, which would benefit sustainable cyber insurance coverage. In terms of cyber risk cause datasets, cyber insurers can use the data to review their insurance products. For example, the data could provide information on which cyber risks have not been sufficiently considered in product design or where improvements are needed. A combination of cyber cause and cybersecurity datasets can help establish uniform definitions to provide greater transparency and clarity. Consistent terminology could lead to a more sustainable cyber market, where cyber insurers make informed decisions about the level of coverage and policyholders understand their coverage (The Geneva Association 2020).

In addition to the cyber insurance community, this research also supports cybersecurity stakeholders. The reviewed literature can be used to provide a contemporary, contextual and categorised summary of available datasets. This supports efficient and timely progress in cyber risk research and is beneficial given the dynamic nature of cyber risks. With the help of the described cybersecurity datasets and the identified information, a comparison of different datasets is possible. The datasets can be used to evaluate the effectiveness of countermeasures in simulated cyberattacks or to test intrusion detection systems.

## Conclusion

In this paper, we conducted a systematic review of studies on cyber risk and cybersecurity databases. We found that most of the datasets are in the field of intrusion detection and machine learning and are used for technical cybersecurity aspects. The available datasets on cyber risks were relatively less represented. Due to the dynamic nature and lack of historical data, assessing and understanding cyber risk is a major challenge for cyber insurance stakeholders. To address this challenge, a greater density of cyber data is needed to support cyber insurers in risk management and researchers with cyber risk-related topics. With reference to ‘Open Science’ FAIR data (Jacobsen et al. 2020), mandatory reporting of cyber incidents could help improve cyber understanding, awareness and loss prevention among companies and insurers. Through greater availability of data, cyber risks can be better understood, enabling researchers to conduct more in-depth research into these risks. Companies could incorporate this new knowledge into their corporate culture to reduce cyber risks. For insurance companies, this would have the advantage that all insurers would have the same understanding of cyber risks, which would support sustainable risk-based pricing. In addition, common definitions of cyber risks could be derived from new data.

The cybersecurity databases summarised and categorised in this research could provide a different perspective on cyber risks that would enable the formulation of common definitions in cyber policies. The datasets can help companies addressing cybersecurity and cyber risk as part of risk management assess their internal cyber posture and cybersecurity measures. The paper can also help improve risk awareness and corporate behaviour, and provides the research community with a comprehensive overview of peer-reviewed datasets and other available datasets in the area of cyber risk and cybersecurity. This approach is intended to support the free availability of data for research. The complete tabulated review of the literature is included in the Supplementary Material.

This work provides directions for several paths of future work. First, there are currently few publicly available datasets for cyber risk and cybersecurity. The older datasets that are still widely used no longer reflect today's technical environment. Moreover, they can often only be used in one context, and the scope of the samples is very limited. It would be of great value if more datasets were publicly available that reflect current environmental conditions. This could help intrusion detection systems to consider current events and thus lead to a higher success rate. It could also compensate for the disadvantages of older datasets by collecting larger quantities of samples and making this contextualisation more widespread. Another area of research may be the integratability and adaptability of cybersecurity and cyber risk datasets. For example, it is often unclear to what extent datasets can be integrated or adapted to existing data. For cyber risks and cybersecurity, it would be helpful to know what requirements need to be met or what is needed to use the datasets appropriately. In addition, it would certainly be helpful to know whether datasets can be modified to be used for cyber risks or cybersecurity. Finally, the ability for stakeholders to identify machine-readable cybersecurity datasets would be useful because it would allow for even clearer delineations or comparisons between datasets. Due to the lack of publicly available datasets, concrete benchmarks often cannot be applied.

## Supplementary Information

Below is the link to the electronic supplementary material.

Supplementary file1 (PDF 334 kb)

Supplementary file1 (DOCX 418 kb)

## Appendix

**Table 2:** Summary of Google datasets

No	Dataset creator	Name of the dataset	Data availability	Year of creation/start year	Description
1	ActionFraud	Cyber Crime Dashboard	Public	2020	Shows cybercrime and fraud reported in the U.K..
2	Carlos E. Jimenez-Gomez	Data Breaches 2004–2017	Public	2018	Includes 270 records and 11 variables of data breaches. The data breaches happened between 2004–2017. Only data breaches with over 30,000 records are considered.
3	Chubb	Chubb Cyber Index	Public	2007	Shows cyber claims for more than two decades. In this dashboard, there is the possibility to get information about different areas regarding claims cost. Furthermore, it is possible to get an overview of claims of different years.
4	CMS	DGDPR Enforcement Tracker	Public	2018	An overview of fines and penalties, which data protection authorities within the EU have imposed under the EU GDPR.
5	DSGVO Portal	DSGVO—Portal	Public	2014	Fines for violations of the GDPR and other data protection laws.
6	Federal Bureau of Investigation	Internet Crime Report 2020	Public	2021	Includes the cyber risk impact situation in the U.S..
7	Government of Canada	No name	Public	2017	Percentage of enterprises impacted by specific types of cybersecurity incidents by the North American Industry Classification System (NAICS) and size of the enterprise.
8	Hiscox	Hisco Cyber Readiness Report 2020	Public	2020	The average cost of all cyberattacks to firms from Europe and the U.S. in 2020, by size, in USD.
9	IBM Security	Cost of a Data Breach Report 2020	Public	2020	Includes the cost of data breaches from 2014 to 2020.
10	Information is beautiful	World's Biggest Data Breaches & Hacks	Public	2004	Selected events over 30,000 records.
11	Ipsos Mori	Cyber Security Breaches Survey	Public	2020	Displays the share of businesses that have had certain outcomes after experiencing a cybersecurity breach or attack in the last 12 months in the U.K. in 2020
12	Kaspersky	Damage Control: The Cost of Security Breaches	Public	2020	Analyses the different data of Kaspersky
13	Marsch—Mircosoft—Global Cyber Risk Perception Survey	Marsch—Mircosoft—Global Cyber Risk Perception Survey	Public	2018	Presents the greatest potential imp.acts to an organisation due to cyber loss scenarios, according to senior executives
14	Mendeley Data	California Data Breach Notification Data	Public	2019	An empirical study of security breach notifications filed in California during 2012–2016.
15	Norton	2019 Cyber Safety Insights Report	Public	2020	A survey of internet users who have experienced an internet crime.
16	Paolo Passeri	Hackmageddon	Access controlled	2011	Overview of collected timelines with a focus on cyberattacks.
17	Pierangelo and Theo	Data Breach Dataset	Public	2020	Consists of 506 data breaches and associated characteristics that affected U.S.-listed companies over a 10-year period from April 2005 to March 2015. The dataset was gathered from the Privacy Rights Clearinghouse (PRC) and then augmented with manual data collection.
18	PwC	2015 Information Security Breaches Survey	Public	2015	Illustrates the ranking of what made a particular security breach incident the worst of the year in the U.K. in 2015.
19	Spy Cloud	Spy Cloud	Private	-	-
20	Willis Towers Watson	Cyber claims analysis report	Public	2020	Uses analysed claims data of Willis Towers Watson to provide specific insight.

## Footnotes

1 Average cost of a breach of more than 50 million records.
