# Supplementary material for: Cyber risk and cybersecurity: a systematic review of data availability
Source: Geneva Pap Risk Insur Issues Pract. 2022 Feb 17;47(3):698–736. doi: 10.1057/s41288-022-00266-6 (PMC8853293; doi:10.1057/s41288-022-00266-6)
Supplement: Supplementary file 1 — Supplementary file1 (PDF 334 kb) [file 41288_2022_266_MOESM1_ESM.pdf]

Dataset Overview

No.	Name of the Dataset	Data Availability	Creation Date	Use cases provided in the academic literature / Feasible cyber insurance application	Place of origin	Short description	Reference includes	Reference
1	ADFA-LD12	Public	2013	Intrusion detection and machine learning / Preventions services for clients	Australia	This dataset uses a Linux operating system.	(Ahmed, Mahmood, and Hu 2016), (Bhattacharya et al. 2020), (Ferrag et al. 2020), (Hindy et al. 2020), (Khraisat et al. 2020), (Sarker et al. 2020)	https://www.unsw.adfa.edu.au/jiankun-hu
2	Aeagan WI-FI Intrusion Dataset 2 (AWID 2)	Public	2016	Intrusion detection and machine learning / Preventions services for clients	Greece	The Aegan Wi-Fi Intrusion Dataset is a publicly available dataset that contained network traffic along with three types of attacks on IEEE 802.11 networks.	(Kasongo and Sun 2020), (Lopez-Martin, Carro, and Sanchez-Esguevillas 2020), (Lee et al. 2020), (Rahman et al. 2021), (Zhou et al. 2020)	https://icsdweb.aegean.gr/awid/
3	Amazon review dataset	Public	2012	Intrusion detection and machine learning / Preventions services for clients	China	The Amazon review dataset comprised data from Amazon. cn until 20 August 2012 and contained 1205,125 ratings from 645,072 users towards 136,785 products.	(Cai, Zhang, and Levi 2019)	(Xu et al. 2013)
4	AndroZoo	Access controlled	2016	Intrusion detection and machine learning / None	Luxembourg	AndroZoo is an increasing collection of Android applications collected from different sources, including the Google Play app. It contained more than 15,400,000 various Android Applications.	(Alazab et al. 2020), (Li and Li 2020), (Bibi et al. 2020)	https://androzoo.uni.lu/
5	Are You You? (RUU dataset)	Access controlled	2009	Behavior modeling / Prevention services for clients	USA	Intrusion detection dataset which contains pc user behavior.	(Al-Mhiqani et al. 2020)	http://ids.cs.columbia.edu/content/ruu.html
6	BoT-IoT	Public	2018	Intrusion detection and machine learning / Assessment of systemic risk	Australia	The BoT-IoT dataset was created by designing a realistic network environment in the Cyber Range Lab at UNSW Canberra. The network environment contained a combination of normal and botnet traffic.	(AlKadi et al. 2019), (Alsamiri and Alsubhi 2019), (Alhowaide, Alsmadi, and Tang 2021), (Biswas and Roy 2021), (Koroniotis, Moustafa, and Sitnikova 2020), (Kumar and Tripathi 2021), (Mauro, Galatro, and Liotta 2020), (Sarker et al. 2020)	https://research.unsw.edu.au/projects/bot-iot-data-set
7	Breach Level Index	Access controlled	N/A	Risk management / Pricing of cyber insurance contracts, contract design (e.g., proposal forms, wordings), prevention services for clients	France	The Breach Level Index tracks security breaches that have become publicly known and also allow organizations to conduct their own risk assessment, as based on a few inputs, it calculates their risk score and the severity of the security breach and summarizes possible actions to reduce the risk score.	(De Giovanni, Leccadito, and Pirra 2020)	https://dis-blog.thalesgroup.com/tag/breach-level-index/
8	Breach Portal	Public	2009	Risk management and modeling / Pricing of cyber insurance contracts, contract design (e.g., proposal forms, wordings), prevention services for clients	USA	Under Title 45 Code of Federal Regulations 164.408 - Notification to the Secretary, any covered health care entity discovering a breach of unsecured protected health information affecting 500 or more individuals must notify the Secretary of DHHS without unreasonable delay and in no case later than 60 days from the discovery of the breach. These data breaches are collected in this database.	(McLeod and Dolezel 2018)	https://ocrportal.hhs.gov/ocr/breach/breach_report.jsf
9	CAIDA 07	Access controlled	2007	Intrusion detection, machine learning and forensic, Pricing of cyber insurance contracts (e.g., business interruption)	USA	This dataset contains approximately one hour of anonymized traffic traces from a DDoS attack on August 4, 2007.	(Aamir and Zaidi 2019), (Agarwal et al. 2021), (Alabdallah and Awad 2018), (Azeez et al. 2019), (Barletta et al. 2020), (Binbusayyis and Vaiyapuri 2019), (Chhabra, Singh, and Singh 2020), (Elmasry, Akbulut, and Zaim 2019), (Gauthama Raman et al. 2020), (Gavel, Raghuvanshi, and Tiwari 2021), (Avila et al. 2021), (Hindy et al. 2020), (Sarker et al. 2020), (Singh and De 2020)	https://www.caida.org/catalog/datasets/ddos-20070804_dataset/
10	CAIDA 08	Access controlled	2008	Intrusion detection and machine learning / Pricing of cyber insurance contracts (e.g., business interruption), prevention services for clients	USA	The dataset contains traces collected from high-speed monitors on a commercial backbone link. The data collection started in April 2008 and ended in January 2019. These data are useful for research on the characteristics of Internet traffic, including application breakdown, security events, geographic and topological distribution, flow volume and duration.	(Aamir and Zaidi 2019), (Avila et al. 2021), (Sarker et al. 2020)	https://www.caida.org/catalog/datasets/passive_dataset/
11	CERT	Public	2014	Behavior activities / Prevention services for clients	USA	The dataset contains activity logs of users that are used for the purpose of validating insider threat detection systems.	(Al-Mhiqani et al. 2020, Sarker et al. 2020)	https://resources.sei.cmu.edu/library/asset-view.cfm?assetid=508099

12	CICIDS2017	Access controlled	2017	Intrusion detection and machine learning / Pricing of cyber insurance contracts by a network model	Canada	The CICIDS2017 dataset contains benign and common cyber attacks. The dataset consists of labelled network flows, including full packet payloads in pcap format, the corresponding profiles and the labelled flows (GeneratedLabelledFlows.zip) and CSV files for machine and deep learning purpose (MachineLearningCSV.zip) are publicly available for researchers.	(Aamir et al. 2021), (Barletta et al. 2020), (Binbusayyis and Vaiyapuri 2019), (D'Hooge et al. 2019), (Elmasry, Akbulut, and Zaim 2019), (Chiba et al. 2019), (Keserwani et al. 2021) (Malik et al. 2020), (Qu et al. 2020), (Javeed, Gao, and Khan 2021), (Monshizadeh et al. 2019), (Li et al. 2020, Lee et al. 2019), (Vinayakumar et al. 2019), (Hindy et al. 2020), (Stojanovic, Hofer-Schmitz, and Kleb 2020, Zhou et al. 2021, Varghese and Muniyal 2021)	https://www.unb.ca/cic/datasets/ids-2017.html
13	CICIDS2018	Access controlled	2018	Intrusion detection and machine learning / Pricing of cyber insurance contracts by a network model	Canada	The dataset includes seven different attack scenarios: Brute-force, Heartbleed, Botnet, DoS, DDoS, Web attacks, and infiltration of the network from inside.	(Chadza, Kyriakopoulos, and Lambotharan 2020), (Atefinia and Ahmadi 2021), (Ahmad and Alsemmeari 2020), (Gavel, Raghuvanshi, and Tiwari 2021), (D'Hooge et al. 2019), (Kilincer, Ertam, and Sengur 2021), (Hindy et al. 2020), (Liu and Lang 2019), (Stojanovic, Hofer-Schmitz, and Kleb 2020)	https://www.unb.ca/cic/datasets/ids-2018.html
14	CIDDS-001	Public	2017	Intrusion detection and machine learning / Prevention services for clients	Germany	The labelled flow-based dataset can be used for training and evaluating network intrusion detection.	(Elmasry, Akbulut, and Zaim 2019), (Oliveira et al. 2021), (Verma and Ranga 2020)	https://www.hs-coburg.de/index.php?id=927
15	CIDDS-002	Public	2017	Intrusion detection and machine learning / Prevention services for clients	Germany	The dataset is containing normal and malicious traffic.	(Elmasry, Akbulut, and Zaim 2019), (Chiba et al. 2019, Verma and Ranga 2020)	https://www.hs-coburg.de/index.php?id=927
16	Contagio	Public	2019	Machine learning and Intrusion detection / Prevention services for clients	-	The Contagio dataset is a collection of malware samples. It contains benign and malicious files.	(Alazab et al. 2020, Ferrag et al. 2020), (Li and Li 2020), (Qiu et al. 2019)	https://www.impactcybertrust.org/dataset_view?idDataset=1273
17	Cost of a cyber incident: Systematic review and cross-validation	Public	2020	Risk management / Pricing of cyber insurance contracts, contract design (e.g., proposal forms, wordings), prevention services for clients, analysis of cyber accumulation risk	USA	As part of cyber risks and cybersecurity, the Cybersecurity & Infrastructure Security Agency has published a study of impacts, costs and losses associated with cyber incidents. The study is intended to assist stakeholders in analyzing cyber risks and cybersecurity. The paper includes approximately 150 articles, industry and government reports, and academic papers that provide historical cost data or derived cost estimates for losses.	Cybersecurity & Infrastructure Security Agency (2020)	https://www.cisa.gov/publication/cost-cyber-incident-systematic-review-and-cross-validation
18	Cost of data breach Report created by Ponemon Institute	Access controlled	N/A	Risk management and modeling / Pricing of cyber insurance contracts, contract design (e.g., proposal forms, wordings)	USA	The Ponemon Institute is known for its annual Cost of Data Breach Study, sponsored by IBM, and its annual Encryption Trends study. In addition, other cyber security relevant topics are covered. The information is published in reports.	(Algarni, Thayananthan, and Malaiya 2021), (Sheehan et al. 2019) (Sheehan et al. 2021)	https://www.ponemon.org/research/ponemon-library/?keywords=cost+of+data
19	CSIC 2010	Public	2010	Intrusion detection and machine learning / Prevention services for clients	Spain	The dataset contains various HHTP protocols request. In this dataset are over 36,000 normal and 25,000 anomalous request.	(Choras and Kozik 2015), (Luo et al. 2020), (Parra et al. 2020)	https://www.tic.itefi.csic.es/dataset/
20	CSI-FBI Survey	Public	2017	Risk Management and modeling / Pricing of cyber insurance, using the information for inclusions and exclusions in contracts	USA	In this dataset, different cyber-attacks and their financial impact were listed.	(Mukhopadhyay et al. 2019)	(Mukhopadhyay et al. 2019)
21	CTIMiner	Public	2019	Intrusion detection and machine learning / Pricing of cyber insurance, trend analysis of events	Republic of Korea	The CTIMiner is an automated dataset generation system, which collects data from publicly available security reports and malware repositories to support cyber threat analysis.	(Kim and Kim 2019), (Sentuna et al. 2021)	https://github.com/dgkim0803/CTIMiner

22	CTU-13	Public	2011	Intrusion detection, machine learning and forensic / Pricing of cyber insurance contracts (e.g., business interruption), claims processing (e.g., forensic), prevention services for clients	Czech Republic	CTU-13 is a dataset of botnet traffic recorded in 2011 at CTU University in the Czech Republic. The dataset consists of thirteen different botnet scenarios.	(Chhabra, Singh, and Singh 2020), (Chowdhury et al. 2017), (Hindy et al. 2020), (Sarker et al. 2020), (Kirubavathi and Anitha 2016), (Sarker et al. 2020)	https://mcfp.weebly.com/the-ctu-13-dataset-a-labeled-dataset-with-botnet-normal-and-background-traffic.html
23	CVE	Public	1999	Risk management, intrusion detection and machine learning / Trend analysis of events, preventions services for clients, network modelling for cyber insurance contracts	USA	The Common Vulnerabilities and Exposures database is one of the most important cybersecurity databases, which contained information about up-to-date vulnerabilities.	(Georgescu, Iancu, and Zurini 2019), (Paté-Cornell et al. 2018), (Subroto and Apriyana 2019)	https://cve.mitre.org/data/downloads/index.html
24	CWE	Public	2006	Intrusion detection and machine learning / Trend analysis of cyber attacks	USA	The Common Weakness Enumeration is a category system for software vulnerabilities and weaknesses. It is supported by a community project with the aim of understanding bugs in software and creating automated tools to identify, fix and prevent them.	(Li, Zhang, et al. 2019)	https://cwe.mitre.org/
25	Cyber Grand Challenge	Public	2016	Intrusion detection and machine learning / Trend analysis of cyber attacks	USA	The Cyber Grand Challenge dataset.	(Li, Zhang, et al. 2019)	https://github.com/CyberGrandChallenge/
26	Cyber Operations Tracker	Public	2005	Risk management / Using the information for inclusions and exclusions in contracts (e.g., cyber war clause), claims processing	USA	Cyber Operations Tracker is a database of publicly known state-sponsored incidents.	Council on foreign Relations (2021)	https://www.cfr.org/cyber-operations/
27	DARPA2000	Public	2000	Attack scenario reconstruction, intrusion detection and machine learning / Pricing of cyber insurance contracts by predictive or probabilistic models	USA	The DARPA 2000 dataset is a multi-stage network attack comprising of two Distributed Denial of Service (DDoS) scenarios.	(Barzegar and Shajari 2018), (Chadza, Kyriakopoulos, and Lambotharan 2020), (AlEroud and Karabatis 2018), (Fan et al. 2018), (Shaukat et al. 2020)	https://www.ll.mit.edu/r-d/datasets/2000-darpa-intrusion-detection-scenario-specific-datasets
28	DARPA98	Public	1998	Intrusion detection and machine learning / Pricing of cyber insurance contracts by predictive or probabilistic models	USA	The DARPA98 dataset is one of the oldest datasets in the history of cybersecurity. The dataset consists of a series of artificial attack injections.	(Liu and Lang 2019), (Bhattacharya et al., 2020), (Feng et al., 2019)	https://www.ll.mit.edu/r-d/datasets
29	DARPA99	Public	1999	Intrusion detection and machine learning / Pricing of cyber insurance contracts by predictive or probabilistic models	USA	The DARPA99 dataset contains emulated network traffic. The dataset consists of three weeks of data, with two weeks with no report and one week with a series of simulated attacks.	(Bouyeddou et al. 2021), (Avila et al. 2021), (Ganeshan and Rodrigues 2020)	https://kdd.ics.uci.edu/
30	Data Breach Investigations Report	Access controlled	2015	Risk management and modeling, Pricing of cyber insurance contracts, prevention services for clients	USA	Verizon publishes the VERIZON Data Breach Investigations Report (DBIR) annually, which provides an overview of the threats that organisations face.	(Algarni, Thayananthan, and Malaiya 2021), (Avila et al. 2021)	https://www.verizon.com/business/resources/reports/dbir/
31	Databreachdb	Public	2021	Risk management and modeling / Pricing of cyber insurance contracts	USA	The database contains about 450 records of major data breaches.	(Neto et al. 2021)	https://databreachdb.com/
32	DEF CON	Public	2013	Intrusion detection, measuring cyber agility (attack and defense) / New insights for preventive services	USA	The DEF CON was created during a "capture the flag (CTF) competition. During this competition, teams try to use the dataset to defend their network while trying to break into opposing networks. The dataset contains only attack traffic and user behavior.	(Avila et al. 2021), (Hindy et al. 2020), (Ferrag et al. 2020), (Mireles et al. 2019)	https://defcon.org/html/links/dc-torrent.html

33	DREBIN	Public	2014	Intrusion detection and machine learning / Trend analysis of events	Germany	The Drebin dataset contains 5,615 malicious Android packages and SHA256 values of 123,453 benign samples.	(Li et al., 2019), (Li and Li 2020), (Varsha et al., 2017), (Arp et al., 2014), (Jahromi et al., 2020), (Tuncer et al., 2020), (Yuan et al., 2020), (Kalutarage, Nguyen, and Shaikh 2017), (Jahromi et al. 2020)	https://www.sec.tu-bs.de/~danarp/drebin/
34	Employment Scam Aegean Dataset	Access controlled	2016	Fraud detection and machine learning / Prevention services for clients	Greece	The Employment Scam Aegean Dataset (EMSCAD) is a publicly available dataset containing 17,880 real-life job ads	(Vidros et al. 2017)	http://emscad.samos.aegean.gr/
35	ENRON	Public	2004	Phishing detection and pattern detection / Trend analysis of events, Pricing of cyber insurance contracts by predictive models	USA	The dataset contains data from about 150 users, mainly from the upper management of the Enron Corporation. This dataset was used to identify complex data leakage patterns and inaccurate sensitive data patterns.	(Ávila et al., 2021), (Miao et al. 2019), (Aassal et al., 2020)	https://www.cs.cmu.edu/~enron/
36	Exploit Prediction Scoring System (EPSS)	Public	2020	Machine Learning / Trend analysis of events, prevention services for clients, pricing of cyber insurance contracts	USA	EPSS is a data-driven vulnerability threat assessment framework, i.e.,	Jacobs et al. (2021)	https://www.first.org/epss/data_stats
37	GTCS (Game Theory and Cyber Security)	Public	2020	Intrusion detection and machine learning / Prevention services for clients	USA	THE GTCS dataset is a labelled dataset, and about 84 network track features have been extracted and used for all benign and intrusive flows. The dataset can be used to assess the performance of multi-stage classifiers.	(Mahfouz et al. 2020)	(Mahfouz et al. 2020)
38	HIMSS	N/A	N/A	Risk management and modeling / Pricing of cyber insurance contracts and insights in healthcare cyber risk data	USA	The dataset is comprised of 65 tables representing technological survey responses from healthcare organizations.	(McLeod and Dolezel 2018)	https://www.himssanalytics.org/resources / ?
39	Identify Theft Center (ITRC) database	Access controlled	N/A	Risk management / Pricing of cyber insurance contracts, prevention services for clients	USA	The ITRC is a not-for-profit organisation established to support and guide consumers, victims, businesses and governments to minimise risk and mitigate the impact of identity compromise and crime. Datasets are released in an annual report.	(Fang et al. 2021)	https://notified.idtheftcenter.org/s/resource
40	ISCXIDS2012	Access controlled	2012	Intrusion detection, machine learning and forensic / Trend analysis of cyber attacks	Canada	The ISCX12 dataset includes normal and anomalous traffic from seven days of network activity. The dataset is also labelled nonanomyized and contains four attack scenarios.	(Aamir and Zaidi, 2019), (Ali and Li, 2019), (Chang et al., 2020), (Chhabra et al., 2020), (Kilincer et al., 2021), (Meira et al., 2020), (Monshizadeh et al., 2019), (Tan et al., 2015), (Avila et al., 2021), (D'hooge et al., 2019), (Ali and Li 2019), (Bhattacharya et al., 2020), (Ferrag et al., 2020), (Hindy et al., 2020), (Sarker et al. 2020), (Sarker et al., 2020), (Amin et al., 2021)	https://www.unb.ca/cic/datasets/ids.html
41	ISOT	Access controlled	2008	Intrusion detection and machine learning / Trend analysis of events, pricing of cyber insurance by a network model	Canada	The ISOT dataset is the combination of existing publicly available malicious and nonmalicious datasets. The malicious traffic originates from the honeynet project and consists of data from the Storm and Waledac botnets.	(Al-Jarrah et al. 2016), (Kirubavathi and Anitha 2016) (Avila et al., 2021)	https://www.uvic.ca/engineering/ece/isot/datasets/
42	ISOT 10	Access controlled	2010	Intrusion detection and machine learning / Trend analysis of events, pricing of cyber insurance by a network model, Prevention services for clients	Canada	This dataset is a combination of malicious and non-malicious datasets created by Information Security and Object Technology. The botnet data for malicious traffic was taken from a Honeynet project. The non-malicious traffic was taken from the Ericsson Research Laboratory and Lawrence Berkeley National Lab	(Aamir and Zaidi, 2019), (Ávila et al., 2021), (Kirubavathi and Anitha, 2016), (Sarker et al., 2020)	https://www.uvic.ca/engineering/ece/isot/datasets/

43	KDD99	Public	1999	Intrusion detection and machine learning / Pricing of cyber insurance contracts by predictive or probabilistic models	USA	The dataset KDD99 was created for the Third International Data Discovery and Data Mining Tools Competition. This database contains a set of data to be examined, including a large number of simulated intrusions in a controlled network environment.	(Alsharafat 2013), (Aamir and Zaidi 2019), (Agrawal, Mohammed, and Fiaidhi 2019), (Alabdallah and Awad 2018), (Azeez et al. 2019), (Barletta et al. 2020), (Binbusayyis and Vaiyapuri 2019), (Chhabra, Singh, and Singh 2020), (Elmasry, Akbulut, and Zaim 2019), (Gauthama Raman et al. 2020), (Gavel, Raghuvanshi, and Tiwari 2021), (Gong et al. 2021), (Avila et al. 2021), (Bhati et al. 2020, Fossaceca, Mazzuchi, and Sarkani 2015), (Keserwani et al. 2021), (Liu and Lang 2019), (Mahbooba et al. 2021), (Mishra and Pandya 2021), (Qu et al. 2020), (Ramaiah et al. 2021), (Raman et al. 2016), (Skrjanc et al. 2018), (Tan et al. 2015), (Velliangiri and Pandey 2020), (Yang et al. 2019), (Ahmed, Mahmood, and Hu 2016), (Adhikari, Morris, and Pan 2018), (Donkal and Verma 2018), (Chattopadhyay, Sen, and Gupta 2018), (Xin et al. 2018), (Agrawal, Mohammed, and Fiaidhi 2019), (Pajouh et al. 2019), (Hajj et al. 2021), (Hong et al. 2020)	https://kdd.ics.uci.edu/
44	KYOTO 2006+ (2015)	Public	2006	Intrusion detection and machine learning / Pricing of cyber insurance contracts (e.g., business interruption)	Japan	The Kyoto dataset is based on real three-year traffic data, which is created using four tools, including honeypots, darknet sensors, e-mail server and web crawler. The dataset contains 24 statistical features, of which 14 features were extracted based on the KDD Cup-99 dataset and ten additional features.	(Gavel et al., 2021), (Avial et al., 2021), (Ferrag et al., 2020), (Uhm and Pak 2021), (Vinayakumar et al., 2019), (Ferrag et al., 2020), (Hindy et al., 2020), (Khan et al. 2020), (Estepa et al. 2020)	http://www.takakura.com/Kyoto_data/
45	LBNL/ICSI	Public	2013	Intrusion detection and machine learning / Pricing of cyber insurance (e.g., business interruption), Trend analysis of events	USA	The dataset consists of traces recorded by a mid-size website and made publicly available in anonymized form and covers a wide spectrum of proportions.	(Avila et al. 2021)	http://www.icir.org/enterprise-tracing/download.html
46	Lexis Nexis Database	Access controlled / Purchase / Free academic source	2020	Risk management and modeling / Information platform for cyber risks	USA	LexisNexis is a company specialising in providing information and technology solutions with international full-text journals, press and business information.	(Arcuri et al. 2020)	https://risk.lexisnexis.com/
47	LITNET-2020	Public	2020	Intrusion detection and machine learning / Analysis of cyber attacks	Lithuania	The dataset is an annotated network benchmark dataset derived from a real academic network. LITNET-2020 represents a real sample of normal and non-attacked network traffic.	(Damasevicius et al. 2020)	https://dataset.litnet.lt/data.php
48	LogoSense	Access controlled	2020	Logo detection and machine learning / Prevention services for clients	Turkey	The LogoSense dataset includes 1530 training patterns and 1979 test patterns. In addition to the 102 brand-specific patterns, we have also included 102 other distraction patterns.	(Bozkir and Aydos 2020)	https://web.cs.hacettepe.edu.tr/~selman/logosense/
49	MACCDC 2012	Public	2012	Attack scenario reconstruction / Claims processing (e.g., forensic)	USA	The MACCDC2012 dataset was created during a National CyberWatch Mid-Atlantic Collegiate Cyber Defense Competition. The dataset covers attacks from scanning/reconnaissance to exploitation.	(Barzegar and Shajari 2018)	https://www.netresec.com/?page=MACCDC
50	MAWI	Public	2010	Intrusion detection and machine learning / Trend analysis of events, analysis of cyber risk accumulation	Canada	The MAWI dataset is real Internet traffic provided by the MAWI Traffic Repository Working Group. The dataset contains traffic from many trans-Pacific links between the Japanese WIDE network and the US.	(Bouyeddou et al., 2020), (Ferrag et al., 2020) (Sarker et al., 2020)	http://www.fukuda-lab.org/mawilab/data.html
51	Microsoft Malware Classification Challenge (BIG 2015)	Public	2015	Machine Learning and malware classification / Pricing of cyber insurance contracts by a network model	USA	The dataset consists of disassembly and bytecode samples from more than 20K malware samples.	(Guo et al. 2020), (Jang, Li, and Sung 2020), (Yuan et al. 2020)	https://www.kaggle.com/c/malware-classication

52	National Vulnerability Database	Public	N/A	Cyber-attack simulator and risk management / Pricing of cyber insurance contracts, contract design (e.g., proposal forms, inclusions, and exclusions in wordings), preventions services for clients	USA	The National Vulnerability Database is the US government's repository for standards-based vulnerability management data presented using the Security Content Automation Protocol. This data allows for the automation of vulnerability management, security measurement and compliance.	(Ashtiani and Azgomi 2014), (Avila et al. 2021), (Johnson et al. 2018), (Sheehan et al. 2019), (Zhang, Ou, and Caragea 2015)	https://nvd.nist.gov/
53	No Name created by a UK-based financial organization	N/A	2018	Taxonomy / Cyber insurance contract design (e.g., standardized definitions)	UK	A real-world dataset of 127 banking Trojans collected from December 2014 to January 2016 by a major UK financial organization. The dataset was used to create a taxonomy of banking Trojans.	(Kiwia et al. 2018)	(Kiwia et al. 2018)
54	No name created by Clusit	Access controlled	2020	Modeling / Insight into a novel model selection measure based on Lorenz zonoids approach	Italy	CLUSIT is an association for the information security industry in Italy, providing awareness, training, professional development and a forum for information exchange on information security issues. The data is provided in the form of an annual report.	(Giudici and Raffinetti 2020)	https://clusit.it/rapporto-clusit/
55	No name created by Fraud Helpdesk (FHD)	Public	2017	Risk management and modeling / Pricing of cyber insurance contracts	Netherlands	The FHD is a Dutch non-profit organization that registers financial crime.	(Junger, Wang, and Schlömer 2020)	https://dans.knaw.nl/en
56	No name created by Husak et al. 2020	Public	2019	Intrusion detection and machine learning / Prevention services for clients	Czech Republic	The dataset consists of the main file with intrusion detection alarms and four auxiliary files with enriched data. The data was taken from a sharing platform.	(Husák et al. 2020)	https://data.mendeley.com/datasets/p6tym3fghz/1
57	No Name created by Laso et al., 2017	Public	2017	Intrusion detection and machine learning / Trend analysis of event, analysis of cyber risk accumulation	France	The dataset represents realistic sensor signals of a cyber-physical subsystem that is affected by actual risks such as anomalies, sabotage, system failures and cyber-attacks.	(Laso, Brosset, and Puentes 2017)	(Laso, Brosset, and Puentes 2017)
58	No Name created by Levi 2017	Public	2016	Risk Management / Trend analysis of events, cyber insurance contract design (e.g., proposal forms)	UK	The article contains different criminal reports from different countries with a focus on cybercrime.	(Levi 2017)	(Levi 2017)
59	No Name created by Moreno et al., 2018	Public	2018	Risk management / Pricing of cyber insurance contracts and insights in chemical and process industry cyber risk data	Italy	The database of 300 safety-related accidents relates to the chemical and process industry. They were collected from different sources.	(Moreno et al. 2018)	(Moreno et al. 2018)
60	No Name created by Pooser, Browne, and Arkhangelska (2018)	Public	2018	Risk management and modeling / Trend analysis of events, pricing of cyber insurance by probabilistic models	USA	The dataset was created by the author to examine the trend in cyber risk identification. They used data from property and casualty insurer.	(Pooser, Browne, and Arkhangelska 2018)	(Pooser, Browne, and Arkhangelska 2018)
61	No Name created by Sovacool	Public	2008	Safety assessment / Assessments of systemic risks, building systemic models	Singapore	The dataset covers data from 1907 to 2007. The dataset was prepared to assess the social and economic costs of energy accidents.	(Sornette, Maillart, and Kröger 2013)	(Sovacool 2008)
62	No name created by Symantec	N/A	2020	Risk management and modeling / Pricing of cyber insurance contracts, Trend analysis of events, parametric modeling, Cooperation between cybersecurity stakeholders and cyber insurers, assessment of cyber risk accumulation	USA	The Symantec dataset contains the number of viruses block on each computer and the number of intrusions blocked on each computer using Symantec software protection an	(Moro 2020)	(Moro 2020)

63	No name is provided by the Center for Machine Learning and Intelligent Systems	Public	2017	Intrusion detection and machine learning / Prevention services for clients	USA	The UCI repository dataset is a combination of nine public IoT datasets, each collected from a different IoT device.	(Habib, Aljarah, and Faris 2020), (Mahdavifar and Ghorbani 2020), (Deng et al. 2019)	https://archive.ics.uci.edu/ml/index.php
64	No name, created by Valeriano and Maness, 2014	Public	2014	Cyber conflicts and pattern description / Using the information to define cyber war clauses, identification of confirmed state sponsored cyber attacks	UK	The dataset contains cyber incidents and cyber disputes between different countries covering the period from 2001 to 2011. The dataset was created with the aim of describing patterns of cyber conflict through cyber interactions between rival states.	(Valeriano and Maness 2014)	https://www.prio.org/jpr/datasets/
65	NSL-KDD 2009	Access controlled	2009	Intrusion detection and machine learning / Prevention services for clients	Canada	The NSL-KDD dataset was developed to counter the KDD-99 criticism, namely that it contains a large amount of redundancy—the number of records in the NSL-KDD. Training and testing dataset is sufficient for most of the anomaly detection as it contains about 150,000 data points.	(Abu Al-Haija and Zein-Sabatto 2020), (Agarwal et al.,2021), (Albahar, Al-Falluji, and Binsawad 2020), (Bhardwaj, Mangat, and Vig 2020), (Binbusayyis et al ., 2019), (D´Hooge et al. 2019), (Dwivedi, Vardhan, and Tripathi 2021), (Elmasry et al., 2018), (Elsaid and Albatati 2020), (Gavel et al., 2021), (Jaber and Ul Rehman 2020), (Ahmad and Alsemmeari 2020), (Almiani et al., 2020), (Ávila et al., 2021), (Kamarudin et al., 2017), (Keserwani et al., 2020), (Kilincer et al., 2021), (Liu and Lang, 2019), (Liu et al., 2021), (Lopez-Martin et al., 2020), (Meira et al., 2020), (Mishra and Pandya 2021), (Pajouh et al., 2019), (Pu et al. 2021), (Rathore et al., 2018), (Verma and Ranga, 2020), (Yang et al., 2019), (Zhang et al., 2019), (Aamir and Zaidi, 2019), (Alabdallah and Awad, 2020), (Chhabra et al., 2020), (Binbusayyis et al ., 2019) (Vinayakumar et al., 2019), (Ferrag et al., 2020), (Hong et al., 2020), (Khraisat et al. 2020), (Mauro et al., 2020), (Moustakidis and Karlsson 2020), (Rahman et al., 2020), (Sarker et al. 2020)	https://www.unb.ca/cic/datasets/nsl.html
66	NVD	Public	N/A	Cyber-attack simulator and risk management / Pricing of cyber insurance contracts, contract design (e.g., proposal forms, inclusions, and exclusions in wordings), preventions services for clients	USA	The National Vulnerability Database is the US government's repository for standards-based vulnerability management data presented using the Security Content Automation Protocol. This data allows for the automation of vulnerability management, security measurement and compliance.	(Astiani et al., (2014), (Ávila et al., 2021), (Johnson et al., 2016), (Sheehan et al., 2019), (Zhang, Ou, and Caragea 2015)	https://nvd.nist.gov/
67	OmniDroid	Public	2019	Intrusion detection and machine learning / Prevention services for clients	Spain	The OmniDroid dataset is a large and comprehensive dataset of features extracted from 22,000 real malware and goodware samples	(Martin, Lara-Cabrera, and Camacho 2019)	https://aida.ii.uam.es/datasets/
68	PKDD-07	Access controlled	2007	Intrusion detection and machine learning / Analysis of cyber-attacks, pricing of cyber insurance contracts (e.g., business interruption)	France	The PKDD07 dataset consists of examples that superficially resemble real attacks but cannot succeed because they are blindly constructed and do not target the correct entities. The following attack types are represented in the dataset: cross-site scripting, SQL injection, LDAP injection, XPATH injection, path traversal, command execution, and server-side include (SSI) injection).	(Avila et al. 2021)	http://www.lirmm.fr/pkdd2007-challenge/
69	PRC Dataset	Public	2005	Machine Learning, risk Management and modeling / Pricing of cyber insurance contracts using copula approaches for modeling cross-sectional dependence of data breach losses	USA	The PRC dataset is publicly available and constantly updated on the databases which contains personal data breaches.	(De Giovanni, Leccadito, and Pirra 2020), (Chen and Fiscus 2018), (Eling and Jung 2018, Fang et al. 2021), (Farkas, Lopez, and Thomas 2021), (Ulven and Wangen 2021), (Bessy-Roland, Boumezoued, and Hillairet 2021).	https://privacyrights.org/data-breaches

70	PREDICT	Access controlled	N/A	Intrusion detection and machine learning / Pricing of cyber insurance contracts, contract design, trend analysis of events, prevention services for clients	USA	The IMPACT Cyber Trust Database supports the global cyber risk research and development community by coordinating and developing opportunities to share real-world data and information between academia, industry and government. Only selected countries have access to the datasets.	(Avila et al. 2021)	https://www.impactcybertrust.org/
71	SAS OPRisk Global Data	N/A	N/A	Risk Management and modeling / Pricing of cyber insurance contracts, Trend analysis of events, assessment for systemic risk	USA	The SAS OPRisk Global Data is a comprehensive database of operational loss information.	(Biener, Eling, and Wirfs 2015), (Eling and Wirfs 2019)	https://www.sas.com/content/dam/SAS/en_us/doc/productbrief/sas-oprisk-global-data-101187.pdf
72	SecurityFocus Vulnerability Database	Public	1999	Intrusion detection and machine learning / Analysis of cyber risk accumulation, trend analysis of events, pricing of cyber insurance contracts by a network model	USA	The database is linked to the BugTraq mailing list and is maintained by Symantec Corporation.	(Johnson et al. 2016)	https://www.securityfocus.com/vulnerabilities
73	SWaT	Public	2016	Intrusion detection and machine learning / Trend analysis of events, prevention services for clients	Singapore	This dataset was created to support research into the design of a secure Cyber-Physical System. In the Context of cyber risk, the data includes attacks from network traffic.	(Farsi, Fanian, and Taghiyarrenani 2019), (Shlomo, Kalech, and Moskovitch 2021)	(Goh et al. 2016)
74	The Hidden costs of cybercrime	Public	2020	Risk management and modeling	USA	The dataset contains different information about cyber crime topics.	(Sheehan et al. 2019)	https://www.mcafee.com/enterprise/en-us/assets/reports/rp-hidden-costs-of-cybercrime.pdf
75	TON_IoT	Public	2020	Intrusion detection and machine learning / Trend analysis of events, pricing of cyber insurance contracts by a network model	Australia	The dataset contains various normal and attack events for different IoT/IIoT services and includes heterogeneous data sources.	(Alsaedi et al., 2020), (Kumar and Tripathi 2021), (Dunn et al., 2020), (Kumar et al. 2021)	https://cloudstor.aarnet.edu.au/plus/s/ds5zW91vdgjEj9i
76	UNSW-NB15	Public	2015	Intrusion detection and machine learning / Trend analysis of events, test data for different event models	Australia	The UNSW-NB15 dataset consists of real and synthetic access activities as normal or attack behavior. The dataset is not anonymized, contains logs from a small network and shows various types of attacks. These include, but are not limited to, DoS, exploits, backdoor attacks, etc.	(Aamir and Zaidi 2019), (Agarwal et al. 2021), (Agrawal, Mohammed, and Fiaidhi 2019), (Al-Omari et al. 2021), (Binbusayyis and Vaiyapuri 2019), (Avila et al. 2021), (Chiba et al. 2019), (Elijah et al. 2019), (Kasongo and Sun 2020), (Keshk et al. 2021), (Kilincer, Ertam, and Sengur 2021), (Koroniotis, Moustafa, and Sitnikova 2020), (Liu and Lang 2019), (Mauro, Galatro, and Liotta 2020), (Manimurugan 2020), (Monshizadeh et al. 2019), (Moustafa et al. 2018, Mwitondi and Zargari 2018), (Stojanovic, Hofer-Schmitz, and Kleb 2020), (Dunn, Moustafa, and Turnbull 2020), (Ferrag et al. 2020)	https://research.unsw.edu.au/projects/unsw-nb15-dataset
77	US Department of Defence	N/A	2018	Event prediction / Pricing of cyber insurance contracts by predictive models, trend analysis of events	USA	The data in the dataset comes from a major operational Computer Security Service Provider (CSSP) for the US Department of Defence. The dataset consists of weekly counts of cyber events over approx—7 years, which were detected by experts.	(Bakdash et al. 2018)	(Bakdash et al., 2018)
78	VERIS Community Database	Public	2013	Risk management and modeling / Pricing of cyber insurance contracts, Trend analysis of events	USA	The database record a range of cybersecurity incident characteristics, including attack mode, actor type, impact, victim type, timeline and prose summaries. The data were also used for the VERZION data breach report.	(Sarabi et al. 2016), (Walker-Roberts et al. 2020)	http://veriscommunity.net/vcdb.html
79	Worldwide DDoS Attacks & Cyber Insights Research Report	Public	2017	Risk management and modeling / Pricing of cyber insurance contracts by probabilistic models, assessment for systemic risk	USA	The dataset contains different information about DDoS attacks and cyber insights.	(Murugesan, Shalinie, and Yang 2018)	https://ns-cdn.neustar.biz/creative_services/biz/neustar/www/resources/whitepapers/it-security/ddos/neustar-2017-worldwide-ddos-attacks-cyber-insights-research-report.pdf
