# Supplementary material for: Cyber risk and cybersecurity: a systematic review of data availability
Source: Geneva Pap Risk Insur Issues Pract. 2022 Feb 17;47(3):698–736. doi: 10.1057/s41288-022-00266-6 (PMC8853293; doi:10.1057/s41288-022-00266-6)
Supplement: Supplementary file 2 — Supplementary file1 (DOCX 418 kb) [file 41288_2022_266_MOESM2_ESM.docx]

**Cyber risk and cybersecurity: a systematic review of data availability**

**Supplementary Table 1: Summary of datasets of cyber causes**

| No. | Name of the Dataset | Data Availability | Creation Date | Use cases provided in the academic literature / Feasible cyber insurance application | Place of origin | Short description | Reference includes | Reference |
| --- | --- | --- | --- | --- | --- | --- | --- | --- |
| 1 | CICIDS2017 | Access controlled | 2017 | Intrusion detection and machine learning / Pricing of cyber insurance contracts by a network model | Canada | The CICIDS2017 dataset contains benign and common cyber attacks. The dataset consists of labelled network flows, including full packet payloads in pcap format, the corresponding profiles and the labelled flows (GeneratedLabelledFlows.zip) and CSV files for machine and deep learning purpose (MachineLearningCSV.zip) are publicly available for researchers. | ([Aamir et al. 2021](#_ENREF_1)), ([Barletta et al. 2020](#_ENREF_28)), ([Binbusayyis and Vaiyapuri 2019](#_ENREF_36)), ([D'Hooge et al. 2019](#_ENREF_48)), ([Elmasry, Akbulut, and Zaim 2019](#_ENREF_58)), ([Chiba et al. 2019](#_ENREF_45)), ([Keserwani et al. 2021](#_ENREF_89)) ([Malik et al. 2020](#_ENREF_113)), ([Qu et al. 2020](#_ENREF_137)), ([Javeed, Gao, and Khan 2021](#_ENREF_83)), ([Monshizadeh et al. 2019](#_ENREF_121)), ([Li et al. 2020](#_ENREF_106), [Lee et al. 2019](#_ENREF_101)), ([Vinayakumar et al. 2019](#_ENREF_162)), ([Hindy et al. 2020](#_ENREF_77)), ([Stojanovic, Hofer-Schmitz, and Kleb 2020](#_ENREF_152), [Zhou et al. 2021](#_ENREF_169), [Varghese and Muniyal 2021](#_ENREF_158)) | https://www.unb.ca/cic/datasets/ids-2017.html |
| 2 | CSI-FBI Survey | Public | 2017 | Risk Management and modeling / Pricing of cyber insurance, using the information for inclusions and exclusions in contracts | U.S. | In this dataset, different cyber-attacks and their financial impact were listed. | ([Mukhopadhyay et al. 2019](#_ENREF_126)) | ([Mukhopadhyay et al. 2019](#_ENREF_126)) |
| 3 | CTIMiner | Public | 2019 | Intrusion detection and machine learning / Pricing of cyber insurance, trend analysis of events | Republic of Korea | The CTIMiner is an automated dataset generation system, which collects data from publicly available security reports and malware repositories to support cyber threat analysis. | ([Kim and Kim 2019](#_ENREF_94)), ([Sentuna et al. 2021](#_ENREF_143)) | <https://github.com/dgkim0803/CTIMiner> |
| 4 | Cyber Operations Tracker | Public | 2005 | Risk management / Using the information for inclusions and exclusions in contracts (e.g., cyber war clause), claims processing | U.S. | Cyber Operations Tracker is a database of publicly known state-sponsored incidents. | Council on foreign Relations (2021) | <https://www.cfr.org/cyber-operations/> |
| 5 | DARPA2000 | Public | 2000 | Attack scenario reconstruction, intrusion detection and machine learning / Pricing of cyber insurance contracts by predictive or probabilistic models | U.S. | The DARPA 2000 dataset is a multi-stage network attack comprising of two Distributed Denial of Service (DDoS) scenarios. | ([Barzegar and Shajari 2018](#_ENREF_29)), ([Chadza, Kyriakopoulos, and Lambotharan 2020](#_ENREF_41)), ([AlEroud and Karabatis 2018](#_ENREF_15)), ([Fan et al. 2018](#_ENREF_61)), ([Shaukat et al. 2020](#_ENREF_144)) | <https://www.ll.mit.edu/r-d/datasets/2000-darpa-intrusion-detection-scenario-specific-datasets> |
| 6 | DEF CON | Public | 2013 | Intrusion detection, measuring cyber agility (attack and defense) / New insights for preventive services | U.S. | The DEF CON was created during a "capture the flag (CTF) competition. During this competition, teams try to use the dataset to defend their network while trying to break into opposing networks. The dataset contains attack traffic and user behavior. | ([Avila et al. 2021](#_ENREF_25)), ([Hindy et al. 2020](#_ENREF_77)), ([Ferrag et al. 2020](#_ENREF_65)), ([Mireles et al. 2019](#_ENREF_119)) | https://defcon.org/html/links/dc-torrent.html |
| 7 | KDD99 | Public | 1999 | Intrusion detection and machine learning / Pricing of cyber insurance contracts by predictive or probabilistic models | U.S. | The dataset KDD99 was created for the Third International Data Discovery and Data Mining Tools Competition. This database contains a set of data to be examined, including a large number of simulated intrusions in a controlled network environment. | ([Alsharafat 2013](#_ENREF_21)), ([Aamir and Zaidi 2019](#_ENREF_2)), ([Agrawal, Mohammed, and Fiaidhi 2019](#_ENREF_6)), ([Alabdallah and Awad 2018](#_ENREF_12)), ([Azeez et al. 2019](#_ENREF_26)), ([Barletta et al. 2020](#_ENREF_28)), ([Binbusayyis and Vaiyapuri 2019](#_ENREF_36)), ([Chhabra, Singh, and Singh 2020](#_ENREF_44)), ([Elmasry, Akbulut, and Zaim 2019](#_ENREF_58)), ([Gauthama Raman et al. 2020](#_ENREF_68)), ([Gavel, Raghuvanshi, and Tiwari 2021](#_ENREF_69)), ([Gong et al. 2021](#_ENREF_73)), ([Avila et al. 2021](#_ENREF_25)), ([Bhati et al. 2020](#_ENREF_32), [Fossaceca, Mazzuchi, and Sarkani 2015](#_ENREF_66)), ([Keserwani et al. 2021](#_ENREF_89)), ([Liu and Lang 2019](#_ENREF_107)), ([Mahbooba et al. 2021](#_ENREF_110)), ([Mishra and Pandya 2021](#_ENREF_120)), ([Qu et al. 2020](#_ENREF_137)), ([Ramaiah et al. 2021](#_ENREF_139)), ([Raman et al. 2016](#_ENREF_140)), ([Skrjanc et al. 2018](#_ENREF_149)), ([Tan et al. 2015](#_ENREF_154)), ([Velliangiri and Pandey 2020](#_ENREF_159)), ([Yang et al. 2019](#_ENREF_166)), ([Ahmed, Mahmood, and Hu 2016](#_ENREF_8)), ([Adhikari, Morris, and Pan 2018](#_ENREF_4)), ([Donkal and Verma 2018](#_ENREF_52)), ([Chattopadhyay, Sen, and Gupta 2018](#_ENREF_42)), ([Xin et al. 2018](#_ENREF_164)), ([Agrawal, Mohammed, and Fiaidhi 2019](#_ENREF_6)), ([Pajouh et al. 2019](#_ENREF_131)), ([Hajj et al. 2021](#_ENREF_76)), ([Hong et al. 2020](#_ENREF_78)) | <https://kdd.ics.uci.edu/> |
| 8 | MACCDC 2012 | Public | 2012 | Attack scenario reconstruction / claims processing (e.g., forensic) | U.S. | The MACCDC2012 dataset was created during a National CyberWatch Mid-Atlantic Collegiate Cyber Defense Competition. The dataset covers attacks from scanning/reconnaissance to exploitation. | ([Barzegar and Shajari 2018](#_ENREF_29)) | <https://www.netresec.com/?page=MACCDC> |
| 9 | Microsoft Malware Classification Challenge (BIG 2015) | Public | 2015 | Machine Learning and malware classification / Pricing of cyber insurance contracts by a network model | U.S. | The dataset consists of disassembly and bytecode samples from more than 20K malware samples. | ([Guo et al. 2020](#_ENREF_74)), ([Jang, Li, and Sung 2020](#_ENREF_82)), ([Yuan et al. 2020](#_ENREF_167)) | [https://www.kaggle.com/c/malware-classication](https://www.kaggle.com/c/malware-classi%1ccation) |
| 10 | No name created by a UK-based financial organization | N/A | 2018 | Taxonomy / Cyber insurance contract design (e.g., standardized definitions) | UK | A real-world dataset of 127 banking Trojans collected from December 2014 to January 2016 by a major UK financial organization. The dataset was used to create a taxonomy of banking Trojans. | ([Kiwia et al. 2018](#_ENREF_96)) | ([Kiwia et al. 2018](#_ENREF_96)) |
| 11 | No name created by Fraud Helpdesk (FHD) | Public | 2017 | Risk management and modeling / Pricing of cyber insurance contracts | Netherlands | The FHD is a Dutch non-profit organization that registers financial crime. | ([Junger, Wang, and Schlömer 2020](#_ENREF_86)) | https://dans.knaw.nl/en |
| 12 | No name created by Levi 2017 | Public | 2016 | Risk Management / Trend analysis of events, cyber insurance contract design (e.g., proposal forms) | UK | The article contains different criminal reports from different countries with a focus on cybercrime. | ([Levi 2017](#_ENREF_103)) | ([Levi 2017](#_ENREF_103)) |
| 13 | No Name created by Sovacool | Public | 2008 | Safety assessment / Assessments of systemic risks, building systemic models | Singapore | The dataset covers data from 1907 to 2007. The dataset was prepared to assess the social and economic costs of energy accidents. | ([Sornette, Maillart, and Kröger 2013](#_ENREF_150)) | ([Sovacool 2008](#_ENREF_151)) |
| 15 | PRC Dataset | Public | 2005 | Machine Learning, risk management and modeling / Pricing of cyber insurance contracts, contract design (e.g., proposal forms, wordings) | U.S. | The PRC dataset is publicly available and constantly updated on the databases which contains personal data breaches. | ([De Giovanni, Leccadito, and Pirra 2020](#_ENREF_50)), ([Chen and Fiscus 2018](#_ENREF_43)), ([Eling and Jung 2018](#_ENREF_56), [Fang et al. 2021](#_ENREF_62)), ([Farkas, Lopez, and Thomas 2021](#_ENREF_63)), ([Ulven and Wangen 2021](#_ENREF_156)), ([Bessy-Roland, Boumezoued, and Hillairet 2021](#_ENREF_30)). | https://privacyrights.org/data-breaches |
| 16 | UNSW-NB15 | Public | 2015 | Intrusion detection and machine learning / Trend analysis of events, test data for different event models | Australia | The UNSW-NB15 dataset consists of real and synthetic access activities as normal or attack behavior. The dataset is not anonymized, contains logs from a small network and shows various types of attacks. These include, but are not limited to, DoS, exploits, backdoor attacks, etc. | ([Aamir and Zaidi 2019](#_ENREF_2)), ([Agarwal et al. 2021](#_ENREF_5)), ([Agrawal, Mohammed, and Fiaidhi 2019](#_ENREF_6)), ([Al-Omari et al. 2021](#_ENREF_11)), ([Binbusayyis and Vaiyapuri 2019](#_ENREF_36)), ([Avila et al. 2021](#_ENREF_25)), ([Chiba et al. 2019](#_ENREF_45)), ([Elijah et al. 2019](#_ENREF_55)), ([Kasongo and Sun 2020](#_ENREF_88)), ([Keshk et al. 2021](#_ENREF_90)), ([Kilincer, Ertam, and Sengur 2021](#_ENREF_93)), ([Koroniotis, Moustafa, and Sitnikova 2020](#_ENREF_97)), ([Liu and Lang 2019](#_ENREF_107)), ([Mauro, Galatro, and Liotta 2020](#_ENREF_116)), ([Manimurugan 2020](#_ENREF_114)), ([Monshizadeh et al. 2019](#_ENREF_121)), ([Moustafa et al. 2018](#_ENREF_124), [Mwitondi and Zargari 2018](#_ENREF_128)), ([Stojanovic, Hofer-Schmitz, and Kleb 2020](#_ENREF_152)), ([Dunn, Moustafa, and Turnbull 2020](#_ENREF_53)), ([Ferrag et al. 2020](#_ENREF_65)) | <https://research.unsw.edu.au/projects/unsw-nb15-dataset> |

.

**Supplementary Table 2: Summary of datasets of cyber impacts**

| No. | Name of the Dataset | Data Availability | Creation Date | Use cases provided in the academic literature / Feasible cyber insurance application | Place of origin | Short description | Reference includes | Reference |
| --- | --- | --- | --- | --- | --- | --- | --- | --- |
| 1 | Breach Level Index | Access controlled | N/A | Risk management / Pricing of cyber insurance contracts, contract design (e.g., proposal forms, wordings), prevention services for clients | France | The Breach Level Index tracks security breaches that have become publicly known and also allow organizations to conduct their own risk assessment, as based on a few inputs, it calculates their risk score and the severity of the security breach and summarizes possible actions to reduce the risk score. | ([De Giovanni, Leccadito, and Pirra 2020](#_ENREF_50)) | <https://dis-blog.thalesgroup.com/tag/breach-level-index/> |
| 2 | Breach Portal | Public | 2009 | Risk management and modeling / Pricing of cyber insurance contracts, contract design (e.g., proposal forms, wordings), prevention services for clients | U.S. | Under Title 45 Code of Federal Regulations 164.408 - Notification to the Secretary, any covered health care entity discovering a breach of unsecured protected health information affecting 500 or more individuals must notify the Secretary of DHHS without unreasonable delay and in no case later than 60 days from the discovery of the breach. These data breaches are collected in this database. | ([McLeod and Dolezel 2018](#_ENREF_117)) | <https://ocrportal.hhs.gov/ocr/breach/breach_report.jsf> |
| 3 | Cost of a cyber incident: Systematic review and cross-validation | Public | 2020 | Risk management / Pricing of cyber insurance contracts, contract design (e.g., proposal forms, wordings), prevention services for clients, analysis of cyber accumulation risk | U.S. | As part of cyber risks and cybersecurity, the Cybersecurity & Infrastructure Security Agency has published a study of impacts, costs and losses associated with cyber incidents. The study is intended to assist stakeholders in analyzing cyber risks and cybersecurity. The paper includes approximately 150 articles, industry and government reports, and academic papers that provide historical cost data or derived cost estimates for losses. | Cybersecurity & Infrastructure Security Agency (2020) | <https://www.cisa.gov/publication/cost-cyber-incident-systematic-review-and-cross-validation> |
| 4 | Cost of data breach Report created by Ponemon Institute | Access controlled | N/A | Risk management and modeling / Pricing of cyber insurance contracts, contract design (e.g., proposal forms, wordings) | U.S. | The Ponemon Institute is known for its annual Cost of Data Breach Study, sponsored by IBM, and its annual Encryption Trends study. In addition, other cyber security relevant topics are covered. The information is published in reports. | ([Algarni, Thayananthan, and Malaiya 2021](#_ENREF_16)), ([Sheehan et al. 2019](#_ENREF_145)) ([Sheehan et al. 2021](#_ENREF_146)) | https://www.ponemon.org/research/ponemon-library/?keywords=cost+of+data |
| 5 | CTIMiner | Public | 2019 | Intrusion detection and machine learning / Pricing of cyber insurance contracts, contract design (e.g., proposal forms, inclusions, and exclusions in wordings) | Republic of Korea | The CTIMiner is an automated dataset generation system, which collects data from publicly available security reports and malware repositories to support cyber threat analysis. | ([Kim and Kim 2019](#_ENREF_94)), ([Sentuna et al. 2021](#_ENREF_143)) | <https://github.com/dgkim0803/CTIMiner> |
| 6 | CVE | Public | 1999 | Risk management, intrusion detection and machine learning / Trend analysis of events, preventions services for clients, network modelling for cyber insurance contracts | U.S. | The Common Vulnerabilities and Exposures database is one of the most important cybersecurity databases, which contained information about up-to-date vulnerabilities. | ([Georgescu, Iancu, and Zurini 2019](#_ENREF_70)), ([Paté-Cornell et al. 2018](#_ENREF_133)), ([Subroto and Apriyana 2019](#_ENREF_153)) | <https://cve.mitre.org/data/downloads/index.html> |
| 7 | Data Breach Investigations Report | Access controlled | 2015 | Risk management and modeling, Pricing of cyber insurance contracts, prevention services for clients | U.S. | Verizon publishes the VERIZON Data Breach Investigations Report (DBIR) annually, which provides an overview of the threats that organizations face. | ([Algarni, Thayananthan, and Malaiya 2021](#_ENREF_16)), ([Avila et al. 2021](#_ENREF_25)) | <https://www.verizon.com/business/resources/reports/dbir/> |
| 8 | Databreachdb | Public | 2021 | Risk management and modeling / Pricing of cyber insurance contracts | U.S. | The database contains about 450 records of major data breaches. | ([Neto et al. 2021](#_ENREF_129)) | <https://databreachdb.com/> |
| 9 | Employment Scam Aegean Dataset | Access controlled | 2016 | Fraud detection and machine learning / Prevention services for clients | Greece | The Employment Scam Aegean Dataset (EMSCAD) is a publicly available dataset containing over 17,000 real-life job ads | ([Vidros et al. 2017](#_ENREF_161)) | <http://emscad.samos.aegean.gr/> |
| 10 | HIMSS | N/A | N/A | Risk management and modeling / Pricing of cyber insurance contracts and insights in healthcare cyber risk data | U.S. | The dataset is comprised of 65 tables representing technological survey responses from healthcare organizations. | ([McLeod and Dolezel 2018](#_ENREF_117)) | https://www.himssanalytics.org/resources / |
| 11 | Identify Theft Center (ITRC) database | Access controlled | N/A | Risk management / Pricing of cyber insurance contracts, prevention services for clients | U.S. | The ITRC is a not-for-profit organisation established to support and guide consumers, victims, businesses and governments to minimise risk and mitigate the impact of identity compromise and crime. Datasets are released in an annual report. | ([Fang et al. 2021](#_ENREF_62)) | https://notified.idtheftcenter.org/s/resource |
| 12 | No Name created by Moreno et al., 2018 | Public | 2018 | Risk management / Pricing of cyber insurance contracts and insights in chemical and process industry cyber risk data | Italy | The database of 300 safety-related accidents relates to the chemical and process industry. They were collected from different sources. | ([Moreno et al. 2018](#_ENREF_122)) | ([Moreno et al. 2018](#_ENREF_122)) |
| 13 | No name created by [Pooser, Browne, and Arkhangelska (2018)](#_ENREF_134) | Public | 2018 | Risk management and modeling / Trend analysis of events, pricing of cyber insurance by probabilistic models | U.S. | The dataset was created by the author to examine the trend in cyber risk identification. They used data from property and casualty insurer. | ([Pooser, Browne, and Arkhangelska 2018](#_ENREF_134)) | ([Pooser, Browne, and Arkhangelska 2018](#_ENREF_134)) |
| 14 | No name, created by Valeriano and Maness, 2014 | Public | 2014 | Cyber conflicts and pattern description / Using the information to define cyber war clauses, identification of confirmed state sponsored cyber attacks | UK | The dataset contains cyber incidents and cyber disputes between different countries covering the period from 2001 to 2011. The dataset was created with the aim of describing patterns of cyber conflict through cyber interactions between rival states. | ([Valeriano and Maness 2014](#_ENREF_157)) | <https://www.prio.org/jpr/datasets/> |
| 15 | National Vulnerability Database | Public | N/A | Cyber-attack simulator and risk management / Pricing of cyber insurance contracts, contract design (e.g., proposal forms, inclusions, and exclusions in wordings), preventions services for clients | U.S. | The National Vulnerability Database is the US government's repository for standards-based vulnerability management data presented using the Security Content Automation Protocol. This data allows for the automation of vulnerability management, security measurement and compliance. | ([Ashtiani and Azgomi 2014](#_ENREF_23)), ([Avila et al. 2021](#_ENREF_25)), ([Johnson et al. 2018](#_ENREF_85)), ([Sheehan et al. 2019](#_ENREF_145)), ([Zhang, Ou, and Caragea 2015](#_ENREF_168)) | <https://nvd.nist.gov/> |
| 16 | No name created by Fraud Helpdesk (FHD) | Public | 2017 | Risk management and modeling / Pricing of cyber insurance contracts by probabilistic models | Netherlands | The FHD is a Dutch non-profit organisation that registers financial crime. | ([Junger, Wang, and Schlömer 2020](#_ENREF_86)); | <https://dans.knaw.nl/en> |
| 17 | SAS OPRisk Global Data | N/A | N/A | Risk Management and modeling / Pricing of cyber insurance contracts, Trend analysis of events, assessment for systemic risk | U.S. | The SAS OPRisk Global Data is a comprehensive database of operational loss information. | ([Biener, Eling, and Wirfs 2015](#_ENREF_35)), ([Eling and Wirfs 2019](#_ENREF_57)) | <https://www.sas.com/content/dam/SAS/en_us/doc/productbrief/sas-oprisk-global-data-101187.pdf> |
| 18 | No name created by Symantec | N/A | 2020 | Risk management and modeling / Pricing of cyber insurance contracts, Trend analysis of events, parametric modeling, Cooperation between cybersecurity stakeholders and cyber insurers, assessment of cyber risk accumulation | U.S. | The Symantec dataset contains the number of viruses block on each computer and the number of intrusions blocked on each computer using Symantec software protection an | ([Moro 2020](#_ENREF_123)) | ([Moro 2020](#_ENREF_123)) |
| 19 | The Hidden costs of cybercrime | Public | 2020 | Risk management and modeling / Pricing of cyber insurance contracts by probabilistic models | U.S. | The dataset contains different information about cybercrime topics. | ([Sheehan et al. 2019](#_ENREF_145)) | [https://www.mcafee.com/enterprise/en-us/assets/reports/rp-hidden-costs-of-cybercrime.pdf](https://www.mcafee.com/enterprise/en-us/about/newsroom/press-releases/press-release.html?news_id=6859bd8c-9304-4147-bdab-32b35457e629) |
| 20 | No name created by Levi 2017 | Public | 2016 | Risk management / Pricing of cyber insurance contracts by probabilistic models | UK | The article contains different criminal reports from different countries with a focus on cybercrime | ([Levi 2017](#_ENREF_103)) | ([Levi 2017](#_ENREF_103)) |
| 21 | VERIS Community Database | Public | 2013 | Risk management and modeling / Pricing of cyber insurance contracts, Trend analysis of events | U.S. | The database records a range of cybersecurity incident characteristics, including attack mode, actor type, impact, victim type, timeline and prose summaries. The data were also used for the VERZION data breach report. | ([Sarabi et al. 2016](#_ENREF_141)), ([Walker-Roberts et al. 2020](#_ENREF_163)) | <http://veriscommunity.net/vcdb.html> |
| 22 | Worldwide DDoS Attacks & Cyber Insights Research Report | Public | 2017 | Risk management and modeling / Pricing of cyber insurance contracts by probabilistic models, assessment for systemic risk | U.S. | The dataset contains different information about DDoS attacks and cyber insights. | ([Murugesan, Shalinie, and Yang 2018](#_ENREF_127)) | <https://ns-cdn.neustar.biz/creative_services/biz/neustar/www/resources/whitepapers/it-security/ddos/neustar-2017-worldwide-ddos-attacks-cyber-insights-research-report.pdf> |
| 23 | No name created by Clusit | Access controlled | 2020 | Modeling / Insight into a novel model selection measure based on Lorenz zonoids approach | Italy | CLUSIT is an association for the information security industry in Italy, providing awareness, training, professional development and a forum for information exchange on information security issues. The data is provided in the form of an annual report. | ([Giudici and Raffinetti 2020](#_ENREF_71)) | <https://clusit.it/rapporto-clusit/> |
| 24 | Lexis Nexis Database | Access controlled / Purchase / Free academic source | 2020 | Risk management and modeling / Information platform for cyber risks | U.S. | LexisNexis is a company specialising in providing information and technology solutions with international full-text journals, press and business information. | ([Arcuri et al. 2020](#_ENREF_22)) | <https://risk.lexisnexis.com/> |
| 25 | US Department of Defense | N/A | 2018 | Event prediction / Pricing of cyber insurance contracts by predictive models | U.S. | The data in the dataset comes from a major operational Computer Security Service Provider (CSSP) for the US Department of Defence. The dataset consists of weekly counts of cyber events over approx. seven years, which were detected by experts. | ([Bakdash et al. 2018](#_ENREF_27)) | ([Bakdash et al. 2018](#_ENREF_27)) |
| 26 | PRC Dataset | Public | 2005 | Machine Learning, risk Management and modeling / Pricing of cyber insurance contracts using copula approaches for modeling cross-sectional dependence of data breach losses | U.S. | The PRC dataset is publicly available and constantly updated on the databases which contains personal data breaches. | ([De Giovanni, Leccadito, and Pirra 2020](#_ENREF_50)), ([Chen and Fiscus 2018](#_ENREF_43)), ([Eling and Jung 2018](#_ENREF_56)), ([Fang et al. 2021](#_ENREF_62)), ([Farkas, Lopez, and Thomas 2021](#_ENREF_63)), ([Ulven and Wangen 2021](#_ENREF_156)), ([Bessy-Roland, Boumezoued, and Hillairet 2021](#_ENREF_30)) | https://privacyrights.org/data-breaches |

**Supplementary Table 3: Summary of datasets of cybersecurity**

| No. | Name of the Dataset | Data Availability | Creation Date | Use cases provided in the academic literature / Feasible cyber insurance application | Place of origin | Short description | Reference includes | Reference |
| --- | --- | --- | --- | --- | --- | --- | --- | --- |
| 1 | Are You You? (RUU dataset) | Access controlled | 2009 | Behavior modeling / Prevention services for clients | U.S. | Intrusion detection dataset which contains pc user behavior. | ([Al-Mhiqani et al. 2020](#_ENREF_10)) | <http://ids.cs.columbia.edu/content/ruu.html> |
| 2 | ADFA-LD12 | Public | 2013 | Intrusion detection and machine learning / Preventions services for clients | Australia | The datasets cover both Linux and Windows. It is designed for evaluation by system call-based Host intrusion detection system. | ([Ahmed, Mahmood, and Hu 2016](#_ENREF_8)), ([Bhattacharya et al. 2020](#_ENREF_33)), ([Ferrag et al. 2020](#_ENREF_65)), ([Hindy et al. 2020](#_ENREF_77)), ([Khraisat et al. 2020](#_ENREF_92)), ([Sarker et al. 2020](#_ENREF_142)) | <https://www.unsw.adfa.edu.au/jiankun-hu> |
| 3 | Aeagan WI-FI Intrusion Dataset 2 (AWID 2) | Public | 2016 | Intrusion detection and machine learning / Preventions services for clients | Greece | The Aegan Wi-Fi Intrusion Dataset is a publicly available dataset that contained network traffic along with three types of attacks on IEEE 802.11 networks. | ([Kasongo and Sun 2020](#_ENREF_88)), ([Lopez-Martin, Carro, and Sanchez-Esguevillas 2020](#_ENREF_108)), ([Lee et al. 2020](#_ENREF_102)), ([Rahman et al. 2021](#_ENREF_138)), ([Zhou et al. 2020](#_ENREF_170)) | <https://icsdweb.aegean.gr/awid/> |
| 4 | Amazon review dataset | Public | 2012 | Intrusion detection and machine learning / Preventions services for clients | China | The Amazon review dataset comprised data from Amazon. cn until 20 August 2012 and contained 1205,125 ratings from 645,072 users towards 136,785 products. | ([Cai, Zhang, and Levi 2019](#_ENREF_40)) | ([Xu et al. 2013](#_ENREF_165)) |
| 5 | AndroZoo | Access controlled | 2016 | Intrusion detection and machine learning / None | Luxembourg | AndroZoo is an increasing collection of Android applications collected from different sources, including the Google Play app. It contained more than 15,400,000 various Android Applications. | ([Alazab et al. 2020](#_ENREF_13)), ([Li and Li 2020](#_ENREF_104)), ([Bibi et al. 2020](#_ENREF_34)) | <https://androzoo.uni.lu/> |
| 6 | BoT-IoT | Public | 2018 | Intrusion detection and machine learning / Assessment of systemic risk | Australia | The BoT-IoT dataset was created by designing a realistic network environment in the Cyber Range Lab at UNSW Canberra. The network environment contained a combination of normal and botnet traffic. | ([AlKadi et al. 2019](#_ENREF_19)), ([Alsamiri and Alsubhi 2019](#_ENREF_20)), ([Alhowaide, Alsmadi, and Tang 2021](#_ENREF_17)), ([Biswas and Roy 2021](#_ENREF_37)), ([Koroniotis, Moustafa, and Sitnikova 2020](#_ENREF_97)), ([Kumar and Tripathi 2021](#_ENREF_99)), ([Mauro, Galatro, and Liotta 2020](#_ENREF_116)), ([Sarker et al. 2020](#_ENREF_142)) | <https://research.unsw.edu.au/projects/bot-iot-data-set> |
| 7 | CAIDA 07 | Access controlled | 2007 | Intrusion detection, machine learning and forensic, Pricing of cyber insurance contracts (e.g., business interruption) | U.S. | This dataset contains approximately one hour of anonymized traffic traces from a DDoS attack on 4th August 2007. | ([Aamir and Zaidi 2019](#_ENREF_2)), ([Agarwal et al. 2021](#_ENREF_5)), ([Alabdallah and Awad 2018](#_ENREF_12)), ([Azeez et al. 2019](#_ENREF_26)), ([Barletta et al. 2020](#_ENREF_28)), ([Binbusayyis and Vaiyapuri 2019](#_ENREF_36)), ([Chhabra, Singh, and Singh 2020](#_ENREF_44)), ([Elmasry, Akbulut, and Zaim 2019](#_ENREF_58)), ([Gauthama Raman et al. 2020](#_ENREF_68)), ([Gavel, Raghuvanshi, and Tiwari 2021](#_ENREF_69)), ([Avila et al. 2021](#_ENREF_25)), ([Hindy et al. 2020](#_ENREF_77)), ([Sarker et al. 2020](#_ENREF_142)), ([Singh and De 2020](#_ENREF_148)) | <https://www.caida.org/catalog/datasets/ddos-20070804_dataset/> |
| 8 | CAIDA 08 | Access controlled | 2008 | Intrusion detection and machine learning / Pricing of cyber insurance contracts (e.g., business interruption), prevention services for clients | U.S. | The dataset contains traces collected from high-speed monitors on a commercial backbone link. The data collection started in April 2008 and ended in January 2019. These data are useful for research on the characteristics of Internet traffic, including application breakdown, security events, geographic and topological distribution, flow volume and duration. | ([Aamir and Zaidi 2019](#_ENREF_2)), ([Avila et al. 2021](#_ENREF_25)), ([Sarker et al. 2020](#_ENREF_142)) | <https://www.caida.org/catalog/datasets/passive_dataset/> |
| 9 | CERT | Public | 2014 | Behavior activities / Prevention services for clients | U.S. | The dataset contains activity logs of users that are used for the purpose of validating insider threat detection systems. | ([Al-Mhiqani et al. 2020](#_ENREF_10), [Sarker et al. 2020](#_ENREF_142)) | <https://resources.sei.cmu.edu/library/asset-view.cfm?assetid=508099> |
| 10 | CICIDS2017 | Access controlled | 2017 | Intrusion detection and machine learning / Pricing of cyber insurance contracts by a network model | Canada | The CICIDS2017 dataset contains benign and common cyber-attacks. The dataset consists of labelled network flows, including full packet payloads in pcap format, the corresponding profiles and the labelled flows (GeneratedLabelledFlows.zip) and CSV files for machine and deep learning purpose (MachineLearningCSV.zip) are publicly available for researchers. | ([Aamir et al. 2021](#_ENREF_1)), ([Barletta et al. 2020](#_ENREF_28)), ([Binbusayyis and Vaiyapuri 2019](#_ENREF_36)), ([D'Hooge et al. 2019](#_ENREF_48)), ([Elmasry, Akbulut, and Zaim 2019](#_ENREF_58)), ([Chiba et al. 2019](#_ENREF_45)), ([Keserwani et al. 2021](#_ENREF_89)) ([Malik et al. 2020](#_ENREF_113)), ([Qu et al. 2020](#_ENREF_137)), ([Javeed, Gao, and Khan 2021](#_ENREF_83)), ([Monshizadeh et al. 2019](#_ENREF_121)), ([Li et al. 2020](#_ENREF_106), [Lee et al. 2019](#_ENREF_101)), ([Vinayakumar et al. 2019](#_ENREF_162)), ([Hindy et al. 2020](#_ENREF_77)), ([Stojanovic, Hofer-Schmitz, and Kleb 2020](#_ENREF_152), [Zhou et al. 2021](#_ENREF_169), [Varghese and Muniyal 2021](#_ENREF_158)) | <https://www.unb.ca/cic/datasets/ids-2017.html> |
| 11 | CICIDS2018 | Access controlled | 2018 | Intrusion detection and machine learning / Pricing of cyber insurance contracts by a network model | Canada | The dataset includes seven different attack scenarios: Brute-force, Heartbleed, Botnet, DoS, DDoS, Web attacks, and infiltration of the network from inside. | ([Chadza, Kyriakopoulos, and Lambotharan 2020](#_ENREF_41)), ([Atefinia and Ahmadi 2021](#_ENREF_24)), ([Ahmad and Alsemmeari 2020](#_ENREF_7)), ([Gavel, Raghuvanshi, and Tiwari 2021](#_ENREF_69)), ([D'Hooge et al. 2019](#_ENREF_48)), ([Kilincer, Ertam, and Sengur 2021](#_ENREF_93)), ([Hindy et al. 2020](#_ENREF_77)), ([Liu and Lang 2019](#_ENREF_107)), ([Stojanovic, Hofer-Schmitz, and Kleb 2020](#_ENREF_152)) | [https://www.unb.ca/cic/datasets/ids-2018.html](https://www.unb.ca/cic/datasets/ids-2018.html%20) |
| 12 | CIDDS-001 | Public | 2017 | Intrusion detection and machine learning / Prevention services for clients | Germany | The labelled flow-based dataset can be used for training and evaluating network intrusion detection. | ([Elmasry, Akbulut, and Zaim 2019](#_ENREF_58)), ([Oliveira et al. 2021](#_ENREF_130)), ([Verma and Ranga 2020](#_ENREF_160)) | https://www.hs-coburg.de/index.php?id=927 |
| 13 | CIDDS-002 | Public | 2017 | Intrusion detection and machine learning / Prevention services for clients | Germany | The dataset is containing normal and malicious traffic. | ([Elmasry, Akbulut, and Zaim 2019](#_ENREF_58)), ([Chiba et al. 2019](#_ENREF_45), [Verma and Ranga 2020](#_ENREF_160)) | https://www.hs-coburg.de/index.php?id=927 |
| 14 | Contagio | Public | 2019 | Machine learning and Intrusion detection / | - | The Contagio dataset is a collection of malware samples. It contains benign and malicious files. | ([Alazab et al. 2020](#_ENREF_13), [Ferrag et al. 2020](#_ENREF_65)), ([Li and Li 2020](#_ENREF_104)), ([Qiu et al. 2019](#_ENREF_136)) | <https://www.impactcybertrust.org/dataset_view?idDataset=1273> |
| 15 | CSIC 2010 | Public | 2010 | Intrusion detection and machine learning / Prevention services for clients | Spain | Contains various HHTP protocols request including over 36,000 normal and 25,000 anomalous request. | ([Choras and Kozik 2015](#_ENREF_46)), ([Luo et al. 2020](#_ENREF_109)), ([Parra et al. 2020](#_ENREF_132)) | <https://www.tic.itefi.csic.es/dataset/> |
| 16 | CTIMiner | Public | 2019 | Intrusion detection and machine learning / Pricing of cyber insurance contracts, contract design (e.g., proposal forms, inclusions, and exclusions in wordings) | Republic of Korea | The CTIMiner is an automated dataset generation system, which collects data from publicly available security reports and malware repositories to support cyber threat analysis. | ([Kim and Kim 2019](#_ENREF_94)), ([Sentuna et al. 2021](#_ENREF_143)) | <https://github.com/dgkim0803/CTIMiner> |
| 17 | CTU-13 | Public | 2011 | Intrusion detection, machine learning and forensic / Pricing of cyber insurance contracts (e.g., business interruption), claims processing (e.g., forensic), prevention services for clients | Czech Republic | CTU-13 is a dataset of botnet traffic recorded in 2011 at CTU University in the Czech Republic. The dataset consists of thirteen different botnet scenarios. | ([Chhabra, Singh, and Singh 2020](#_ENREF_44)), ([Chowdhury et al. 2017](#_ENREF_47)), ([Hindy et al. 2020](#_ENREF_77)), ([Sarker et al. 2020](#_ENREF_142)), ([Kirubavathi and Anitha 2016](#_ENREF_95)), ([Sarker et al. 2020](#_ENREF_142)) | <https://mcfp.weebly.com/the-ctu-13-dataset-a-labeled-dataset-with-botnet-normal-and-background-traffic.html> |
| 18 | CVE | Public | 1999 | Risk management, intrusion detection and machine learning / Trend analysis of events, preventions services for clients, network modelling for cyber insurance contracts | U.S. | The Common Vulnerabilities and Exposures database is one of the most important cybersecurity databases, which contained information about up-to-date vulnerabilities. | ([Georgescu, Iancu, and Zurini 2019](#_ENREF_70)), ([Paté-Cornell et al. 2018](#_ENREF_133)), ([Subroto and Apriyana 2019](#_ENREF_153)) | <https://cve.mitre.org/data/downloads/index.html> |
| 19 | CWE | Public | 2006 | Intrusion detection and machine learning / Trend analysis of cyber attacks | U.S. | The Common Weakness Enumeration is a category system for software vulnerabilities and weaknesses. It is supported by a community project with the aim of understanding bugs in software and creating automated tools to identify, fix and prevent them. | ([Li et al. 2019](#_ENREF_105)) | <https://cwe.mitre.org/> |
| 20 | Cyber Grand Challenge | Public | 2016 | Intrusion detection and machine learning / Trend analysis of cyber attacks | U.S. | Contains the data collected during the Cyber Grand Challenge. | ([Li et al. 2019](#_ENREF_105)) | <https://github.com/CyberGrandChallenge/> |
| 21 | DARPA Intrusion Detection Evaluation | Public | 1999 | Attack scenario reconstruction, intrusion detection and machine learning / Pricing of cyber insurance contracts by predictive or probabilistic models | U.S. | The DARPA2000 dataset has two similar attack scenarios. The aim of the dataset is to start a DDoS attack. | (Chattopadhyay et al. 2018), ( Hemo et al., 2020), (Ahmed et al., 2016), (Xin et al., 2018), (Sarker et al., 2020) | <https://www.ll.mit.edu/r-d/datasets/1999-darpa-intrusion-detection-evaluation-dataset> |
| 22 | DARPA2000 | Public | 2000 | Attack scenario reconstruction, intrusion detection and machine learning / Pricing of cyber insurance contracts by predictive or probabilistic models | U.S. | The DARPA 2000 dataset is a multi-stage network attack comprising of two Distributed Denial of Service (DDoS) scenarios | (Barzegar and Shajari, 2018), (Chadza et al., 2020), Aleroud and Karabatis 2018), (Fan et al. 2018), (Shaukat et al., 2020) | <https://www.ll.mit.edu/r-d/datasets/2000-darpa-intrusion-detection-scenario-specific-datasets> |
| 23 | DARPA98 | Public | 1998 | Intrusion detection and machine learning / Pricing of cyber insurance contracts by predictive or probabilistic models | U.S. | The DARPA98 dataset is one of the oldest datasets in the history of cybersecurity. The dataset consists of a series of artificial attack injections. | (Liu and Lang 2019), (Bhattacharya et al., 2020), | <https://www.ll.mit.edu/r-d/datasets> |
| 24 | DARPA99 | Public | 1999 | Intrusion detection and machine learning / Pricing of cyber insurance contracts by predictive or probabilistic models | U.S. | The DARPA99 dataset contains emulated network traffic. The dataset consists of three weeks of data, with two weeks with no report and one week with a series of simulated attacks. | ([Bouyeddou et al. 2021](#_ENREF_38)), ([Avila et al. 2021](#_ENREF_25)), ([Ganeshan and Rodrigues 2020](#_ENREF_67)) | <https://kdd.ics.uci.edu/> |
| 25 | DEF CON | Public | 2013 | Intrusion detection, measuring cyber agility (attack and defense) / New insights for preventive services | U.S. | The DEF CON was created during a "capture the flag (CTF) competition. During this competition, teams try to use the dataset to defend their network while trying to break into opposing networks. The dataset contains only attack traffic and user behaviour | (Ávila et al., 2021), (Mireless et al., 2019), (Ferrag et al., 2020), (Hindy et al., 2020) | <https://www.defcon.org/> |
| 26 | DREBIN | Public | 2014 | Intrusion detection and machine learning / Trend analysis of events | Germany | The Drebin dataset contains 5,615 malicious Android packages and SHA256 values of 123,453 benign samples. | (Li et al., 2019), (Li and Li 2020), (Varsha et al., 2017), (Arp et al., 2014), (Jahromi et al., 2020), (Tuncer et al., 2020), (Yuan et al., 2020), ([Kalutarage, Nguyen, and Shaikh 2017](#_ENREF_87)), ([Jahromi et al. 2020](#_ENREF_81)) | <https://www.sec.tu-bs.de/~danarp/drebin/> |
| 27 | ENRON | Public | 2004 | Phishing detection and pattern detection / Trend analysis of events, Pricing of cyber insurance contracts by predictive models | U.S. | The dataset contains data from about 150 users, mainly from the upper management of the Enron Corporation. This dataset was used to identify complex data leakage patterns and inaccurate sensitive data patterns. | (Ávila et al., 2021), ([Miao et al. 2019](#_ENREF_118)), (Aassal et al., 2020) | <https://www.cs.cmu.edu/~enron/> |
| 28 | Exploit Prediction Scoring System (EPSS) | Public | 2020 | Machine Learning / Trend analysis of events, prevention services for clients, pricing of cyber insurance contracts | U.S. | EPSS is a data-driven vulnerability threat assessment framework, i.e.,  the likelihood that a vulnerability will be exploited in an open environment within the first 12 months of its disclosure. | Jacobs et al. (2021) | <https://www.first.org/epss/data_stats> |
| 29 | GTCS (Game Theory and Cyber Security) | Public | 2020 | Intrusion detection and machine learning / Prevention services for clients | U.S. | THE GTCS dataset is a labelled dataset, and about 84 network track features have been extracted and used for all benign and intrusive flows. The dataset can be used to assess the performance of multi-stage classifiers. | ([Mahfouz et al. 2020](#_ENREF_112)) | ([Mahfouz et al. 2020](#_ENREF_112)) |
| 30 | ISCXIDS2012 | Access controlled | 2012 | Intrusion detection, machine learning and forensic / Trend analysis of cyber attacks | Canada | The ISCX12 dataset includes normal and anomalous traffic from seven days of network activity. The dataset is also labelled non-anonymized and contains four attack scenarios. | (Aamir and Zaidi, 2019), (Ali and Li, 2019), (Chang et al., 2020), (Chhabra et al., 2020), (Kilincer et al., 2021), (Meira et al., 2020), (Monshizadeh et al., 2019), (Tan et al., 2015), (Avila et al., 2021), (D'hooge et al., 2019), ([Ali and Li 2019](#_ENREF_18)), (Bhattacharya et al., 2020), (Ferrag et al., 2020), (Hindy et al., 2020), (Sarker et al. 2020), (Sarker et al., 2020), (Amin et al., 2021) | <https://www.unb.ca/cic/datasets/ids.html> |
| 31 | ISOT | Access controlled | 2008 | Intrusion detection and machine learning / Trend analysis of events, pricing of cyber insurance by a network model | Canada | The ISOT dataset is the combination of existing publicly available malicious and nonmalicious datasets. The malicious traffic originates from the honeynet project and consists of data from the Storm and Waledac botnets. | ([Al-Jarrah et al. 2016](#_ENREF_9)), (Kirubavathi and Anitha 2016) (Avila et al., 2021) | <https://www.uvic.ca/engineering/ece/isot/datasets/> |
| 32 | ISOT 10 | Access controlled | 2010 | Intrusion detection and machine learning / Trend analysis of events, pricing of cyber insurance by a network model, Prevention services for clients | Canada | This dataset is a combination of malicious and non-malicious datasets created by Information Security and Object Technology. The botnet data for malicious traffic was taken from a Honeynet project. The non-malicious traffic was taken from the Ericsson Research Laboratory and Lawrence Berkeley National Lab | (Aamir and Zaidi, 2019), (Ávila et al., 2021), (Kirubavathi and Anitha, 2016), (Sarker et al., 2020) | <https://www.uvic.ca/engineering/ece/isot/datasets/> |
| 33 | KDD99 | Public | 1999 | Intrusion detection and machine learning / Pricing of cyber insurance contracts by predictive or probabilistic models | U.S. | The dataset KDD99 was created for the Third International Data Discovery and Data Mining Tools Competition. This database contains a set of data to be examined, including a large number of simulated intrusions in a controlled network environment. | ([Alsharafat 2013](#_ENREF_21)), ([Aamir and Zaidi 2019](#_ENREF_2)), ([Agrawal, Mohammed, and Fiaidhi 2019](#_ENREF_6)), ([Alabdallah and Awad 2018](#_ENREF_12)), ([Azeez et al. 2019](#_ENREF_26)), ([Barletta et al. 2020](#_ENREF_28)), ([Binbusayyis and Vaiyapuri 2019](#_ENREF_36)), ([Chhabra, Singh, and Singh 2020](#_ENREF_44)), ([Elmasry, Akbulut, and Zaim 2019](#_ENREF_58)), ([Gauthama Raman et al. 2020](#_ENREF_68)), ([Gavel, Raghuvanshi, and Tiwari 2021](#_ENREF_69)), ([Gong et al. 2021](#_ENREF_73)), ([Avila et al. 2021](#_ENREF_25)), ([Bhati et al. 2020](#_ENREF_32), [Fossaceca, Mazzuchi, and Sarkani 2015](#_ENREF_66)), ([Keserwani et al. 2021](#_ENREF_89)), ([Liu and Lang 2019](#_ENREF_107)), ([Mahbooba et al. 2021](#_ENREF_110)), ([Mishra and Pandya 2021](#_ENREF_120)), ([Qu et al. 2020](#_ENREF_137)), ([Ramaiah et al. 2021](#_ENREF_139)), ([Raman et al. 2016](#_ENREF_140)), ([Skrjanc et al. 2018](#_ENREF_149)), ([Tan et al. 2015](#_ENREF_154)), ([Velliangiri and Pandey 2020](#_ENREF_159)), ([Yang et al. 2019](#_ENREF_166)), ([Ahmed, Mahmood, and Hu 2016](#_ENREF_8)), ([Adhikari, Morris, and Pan 2018](#_ENREF_4)), ([Donkal and Verma 2018](#_ENREF_52)), ([Chattopadhyay, Sen, and Gupta 2018](#_ENREF_42)), ([Xin et al. 2018](#_ENREF_164)), ([Agrawal, Mohammed, and Fiaidhi 2019](#_ENREF_6)), ([Pajouh et al. 2019](#_ENREF_131)), ([Hajj et al. 2021](#_ENREF_76)), ([Hong et al. 2020](#_ENREF_78)) | <https://kdd.ics.uci.edu/> |
| 34 | KYOTO 2006+ (2015) | Public | 2006 | Intrusion detection and machine learning / Pricing of cyber insurance contracts (e.g., business interruption) | Japan | The Kyoto dataset is based on real three-year traffic data, which is created using four tools, including honeypots, darknet sensors, e-mail server and web crawler. The dataset contains 24 statistical features, of which 14 features were extracted based on the KDD Cup-99 dataset and ten additional features. | (Gavel et al., 2021), (Avial et al., 2021), (Ferrag et al., 2020), ([Uhm and Pak 2021](#_ENREF_155)), (Vinayakumar et al., 2019), (Ferrag et al., 2020), (Hindy et al., 2020), ([Khan et al. 2020](#_ENREF_91)), ([Estepa et al. 2020](#_ENREF_60)) | <http://www.takakura.com/Kyoto_data/> |
| 35 | No Name created by Laso et al., 2017 | Public | 2017 | Intrusion detection and machine learning / Trend analysis of event, analysis of cyber risk accumulation | France | The dataset represents realistic sensor signals of a cyber-physical subsystem that is affected by actual risks such as anomalies, sabotage, system failures and cyber-attacks. | ([Laso, Brosset, and Puentes 2017](#_ENREF_100)) | (Laso, Brosset, and Puentes 2017) |
| 36 | LBNL/ICSI | Public | 2013 | Intrusion detection and machine learning / Pricing of cyber insurance (e.g., business interruption), Trend analysis of events | U.S. | The dataset consists of traces recorded by a mid-size website and made publicly available in anonymized form and covers a wide spectrum of proportions. | ([Avila et al. 2021](#_ENREF_25)) | <http://www.icir.org/enterprise-tracing/download.html> |
| 37 | LITNET-2020 | Public | 2020 | Intrusion detection and machine learning / Analysis of cyber attacks | Lithuania | The dataset is an annotated network benchmark dataset derived from a real academic network. LITNET-2020 represents a real sample of normal and non-attacked network traffic. | ([Damasevicius et al. 2020](#_ENREF_49)) | https://dataset.litnet.lt/data.php |
| 38 | LogoSense | Access controlled | 2020 | Logo detection and machine learning / Prevention services for clients | Turkey | The LogoSense dataset includes 1530 training patterns and 1979 test patterns. In addition to the 102 brand-specific patterns, they have also included 102 other distraction patterns. | ([Bozkir and Aydos 2020](#_ENREF_39)) | <https://web.cs.hacettepe.edu.tr/~selman/logosense/> |
| 39 | MACCDC 2012 | Public | 2012 | Attack scenario reconstruction / Claims processing (e.g., forensic) | U.S. | The MACCDC2012 dataset was created during a National CyberWatch Mid-Atlantic Collegiate Cyber Defense Competition. The dataset covers attacks from scanning/reconnaissance to exploitation. | ([Barzegar and Shajari 2018](#_ENREF_29)) | <https://www.netresec.com/?page=MACCDC> |
| 40 | MAWI | Public | 2010 | Intrusion detection and machine learning / Trend analysis of events, analysis of cyber risk accumulation | Canada | The MAWI dataset is real Internet traffic provided by the MAWI Traffic Repository Working Group. The dataset contains traffic from many trans-Pacific links between the Japanese WIDE network and the US. | (Bouyeddou et al., 2020), (Ferrag et al., 2020) (Sarker et al., 2020) | <http://www.fukuda-lab.org/mawilab/data.html> |
| 41 | Microsoft Malware Classification Challenge (BIG 2015) | Public | 2015 | Machine Learning and malware classification / Pricing of cyber insurance contracts by a network model | U.S. | The dataset consists of disassembly and bytecode samples from more than 20K malware samples. | ([Guo et al. 2020](#_ENREF_74)), ([Jang, Li, and Sung 2020](#_ENREF_82)), ([Yuan et al. 2020](#_ENREF_167)) | [https://www.kaggle.com/c/malware-classication](https://www.kaggle.com/c/malware-classi%1ccation) |
| 42 | No Name created by Moreno et al., 2018 | Public | 2018 | Risk management and modeling / Pricing of cyber insurance contracts, Trend analysis of events, parametric modeling, Cooperation between stakeholders and insurers | Italy | The database of 300 safety-related accidents relates to the chemical and process industry. They were collected from different sources. | ([Moreno et al. 2018](#_ENREF_122)) | ([Moreno et al. 2018](#_ENREF_122)) |
| 43 | NSL-KDD 2009 | Access controlled | 2009 | Intrusion detection and machine learning / Prevention services for clients | Canada | The NSL-KDD dataset was developed to counter the KDD-99 criticism, namely that it contains a large amount of redundancy—the number of records in the NSL-KDD. Training and testing dataset is sufficient for most of the anomaly detection as it contains about 150,000 data points. | ([Abu Al-Haija and Zein-Sabatto 2020](#_ENREF_3)), (Agarwal et al.,2021), ([Albahar, Al-Falluji, and Binsawad 2020](#_ENREF_14)), ([Bhardwaj, Mangat, and Vig 2020](#_ENREF_31)), (Binbusayyis et al ., 2019), (D´Hooge et al. 2019), ([Dwivedi, Vardhan, and Tripathi 2021](#_ENREF_54)), (Elmasry et al., 2018), ([Elsaid and Albatati 2020](#_ENREF_59)), (Gavel et al., 2021), ([Jaber and Ul Rehman 2020](#_ENREF_80)), ([Ahmad and Alsemmeari 2020](#_ENREF_7)), (Almiani et al., 2020), (Ávila et al., 2021), (Kamarudin et al., 2017), (Keserwani et al., 2020), (Kilincer et al., 2021), (Liu and Lang, 2019), (Liu et al., 2021), (Lopez-Martin et al., 2020), (Meira et al., 2020), (Mishra and Pandya 2021), (Pajouh et al., 2019), ([Pu et al. 2021](#_ENREF_135)), (Rathore et al., 2018), (Verma and Ranga, 2020), (Yang et al., 2019), (Zhang et al., 2019), (Aamir and Zaidi, 2019), (Alabdallah and Awad, 2020), (Chhabra et al., 2020), (Binbusayyis et al ., 2019) (Vinayakumar et al., 2019), (Ferrag et al., 2020), (Hong et al., 2020), (Khraisat et al. 2020), (Mauro et al., 2020), ([Moustakidis and Karlsson 2020](#_ENREF_125)), (Rahman et al., 2020), (Sarker et al. 2020) | <https://www.unb.ca/cic/datasets/nsl.html> |
| 44 | NVD | Public | N/A | Cyber-attack simulator and risk management / Pricing of cyber insurance contracts, contract design (e.g., proposal forms, inclusions, and exclusions in wordings), preventions services for clients | U.S. | The National Vulnerability Database is the US government's repository for standards-based vulnerability management data presented using the Security Content Automation Protocol. This data allows for the automation of vulnerability management, security measurement and compliance. | (Astiani et al., (2014), (Ávila et al., 2021), (Johnson et al., 2016), (Sheehan et al., 2019), ([Zhang, Ou, and Caragea 2015](#_ENREF_168)) | <https://nvd.nist.gov/> |
| 45 | OmniDroid | Public | 2019 | Intrusion detection and machine learning / Prevention services for clients | Spain | The OmniDroid dataset is a large and comprehensive dataset of features extracted from 22,000 real malware and goodware samples | ([Martin, Lara-Cabrera, and Camacho 2019](#_ENREF_115)) | <https://aida.ii.uam.es/datasets/> |
| 46 | PKDD-07 | Access controlled | 2007 | Intrusion detection and machine learning / Analysis of cyber-attacks, pricing of cyber insurance contracts (e.g., business interruption) | France | The PKDD07 dataset consists of examples that superficially resemble real attacks but cannot succeed because they are blindly constructed and do not target the correct entities. The following attack types are represented in the dataset: cross-site scripting, SQL injection, LDAP injection, XPATH injection, path traversal, command execution, and server-side include (SSI) injection). | ([Avila et al. 2021](#_ENREF_25)) | <http://www.lirmm.fr/pkdd2007-challenge/> |
| 47 | PREDICT | Access controlled | N/A | Intrusion detection and machine learning / Pricing of cyber insurance contracts, contract design, trend analysis of events, prevention services for clients | U.S. | The IMPACT Cyber Trust Database supports the global cyber risk research and development community by coordinating and developing opportunities to share real-world data and information between academia, industry and government. Only selected countries have access to the datasets. | ([Avila et al. 2021](#_ENREF_25)) | <https://www.impactcybertrust.org/> |
| 48 | SecurityFocus Vulnerability Database | Public | 1999 | Intrusion detection and machine learning / Analysis of cyber risk accumulation, trend analysis of events, pricing of cyber insurance contracts by a network model | U.S. | The database is linked to the BugTraq mailing list and is maintained by Symantec Corporation. | ([Johnson et al. 2016](#_ENREF_84)) | <https://www.securityfocus.com/vulnerabilities> |
| 49 | TON_IoT | Public | 2020 | Intrusion detection and machine learning / Trend analysis of events, pricing of cyber insurance contracts by a network model | Australia | The dataset contains various normal and attack events for different IoT/IIoT services and includes heterogeneous data sources. | (Alsaedi et al., 2020), ([Kumar and Tripathi 2021](#_ENREF_99)), (Dunn et al., 2020), ([Kumar et al. 2021](#_ENREF_98)) | <https://cloudstor.aarnet.edu.au/plus/s/ds5zW91vdgjEj9i> |
| 50 | No name is provided by the Center for Machine Learning and Intelligent Systems | Public | 2017 | Intrusion detection and machine learning / Prevention services for clients | U.S. | The UCI repository dataset is a combination of nine public IoT datasets, each collected from a different IoT device. | ([Habib, Aljarah, and Faris 2020](#_ENREF_75)), ([Mahdavifar and Ghorbani 2020](#_ENREF_111)), ([Deng et al. 2019](#_ENREF_51)) | <https://archive.ics.uci.edu/ml/index.php> |
| 51 | US Department of Defence | N/A | 2018 | Event prediction / Pricing of cyber insurance contracts by predictive models, trend analysis of events | U.S. | The data in the dataset comes from a major operational Computer Security Service Provider (CSSP) for the US Department of Defence. The dataset consists of weekly counts of cyber events over approx. seven years, which were detected by experts. | ([Bakdash et al. 2018](#_ENREF_27)) | (Bakdash et al., 2018) |
| 52 | PRC Dataset | Public | 2005 | Machine Learning, risk Management and modeling / Pricing of cyber insurance contracts using copula approaches for modeling cross-sectional dependence of data breach losses | U.S. | The PRC dataset is publicly available and constantly updated on the databases which contain personal data breaches. | (De Giovanni et al., 2020), (Chen and Fiscus, 2018) (Eling and Jung, 2018), (Fang et al., 2021), (Farkas et al., 2021), (Ulven and Wangen, 2021), (Bessy-Roland et al., 202). | <https://privacyrights.org/data-breaches> |
| 53 | SWaT | Public | 2016 | Intrusion detection and machine learning / Trend analysis of events, prevention services for clients | Singapore | This dataset was created to support research into the design of a secure Cyber-Physical System. In the Context of cyber risk, the data includes attacks from network traffic. | ([Farsi, Fanian, and Taghiyarrenani 2019](#_ENREF_64)), ([Shlomo, Kalech, and Moskovitch 2021](#_ENREF_147)) | ([Goh et al. 2016](#_ENREF_72)) |
| 54 | UNSW-NB15 | Public | 2015 | Intrusion detection and machine learning / Trend analysis of events, test data for different event models | Australia | The UNSW-NB15 dataset consists of real and synthetic access activities as normal or attack behavior. The dataset is not anonymized, contains logs from a small network and shows various types of attacks. These include, but are not limited to, DoS, exploits, backdoor attacks, etc. | (Aamir and Zaidi, 2019), (Agrarwal et al., 2021), (Al-Omari et al., 2021), (Alkadi et al., 2019), (Binbusayyis et al ., 2019), (Gauthama Raman et al., 2019), (Ávila et al., 2021), (Chiba et al., 2019), (Elijah et al., 2019), (Kasongo and Sun, 2020), (Keshk et al., 2021), (Kilincer et al., 2021), (Koroniotis et al., 2020), (Liu and Lang, 2019), (Mauro et al., 2020), (Monshizadeh et al., 2019), (Moustafa et al., 2018), (Verma and Ranga, 2020), (Agrawal et al. 2019), (Binbusayyis et al., 2019), (Vinayakumar et al., 2019), (Ferrag et al., 2020), (Keshk et al., 2020), (Manimurugan 2020), (Sarker et al., 2020), (Stojanovic et al., 2020) (Dunn et al., 2020), (Mwitondi and Zargari, 2018) | <https://research.unsw.edu.au/projects/unsw-nb15-dataset> |
| 55 | No name created by Husak et al. 2020 | Public | 2019 | Intrusion detection and machine learning / Prevention services for clients | Czech Republic | The dataset consists of the main file with intrusion detection alarms and four auxiliary files with enriched data. The data was taken from a sharing platform. | ([Husák et al. 2020](#_ENREF_79)) | <https://data.mendeley.com/datasets/p6tym3fghz/1> |

**Bibliography**

Aamir, M., S. S. H. Rizvi, M. A. Hashmani, M. Zubair, and J. Ahmad. 2021. "Machine Learning Classification of Port Scanning and DDoS Attacks: A Comparative Analysis." *Mehran University Research Journal of Engineering and Technology* 40 (1):215-229. doi: 10.22581/muet1982.2101.19.

Aamir, M., and S. M. A. Zaidi. 2019. "DDoS attack detection with feature engineering and machine learning: the framework and performance evaluation." *International Journal of Information Security* 18 (6):761-785. doi: 10.1007/s10207-019-00434-1.

Abu Al-Haija, Q., and S. Zein-Sabatto. 2020. "An Efficient Deep-Learning-Based Detection and Classification System for Cyber-Attacks in IoT Communication Networks." *Electronics* 9 (12):26. doi: 10.3390/electronics9122152.

Adhikari, U., T. H. Morris, and S. Y. Pan. 2018. "Applying Hoeffding Adaptive Trees for Real-Time Cyber-Power Event and Intrusion Classification." *Ieee Transactions on Smart Grid* 9 (5):4049-4060. doi: 10.1109/tsg.2017.2647778.

Agarwal, A., P. Sharma, M. Alshehri, A. A. Mohamed, and O. Alfarraj. 2021. "Classification model for accuracy and intrusion detection using machine learning approach." *Peerj Computer Science*:22. doi: 10.7717/peerj-cs.437.

Agrawal, A., S. Mohammed, and J. Fiaidhi. 2019. "ENSEMBLE TECHNIQUE FOR INTRUDER DETECTION IN NETWORK TRAFFIC." *International Journal of Security and Its Applications* 13 (3):1-8. doi: 10.33832/ijsia.2019.13.3.01.

Ahmad, I., and R. A. Alsemmeari. 2020. "Towards Improving the Intrusion Detection through ELM (Extreme Learning Machine)." *Cmc-Computers Materials & Continua* 65 (2):1097-1111. doi: 10.32604/cmc.2020.011732.

Ahmed, M., A. N. Mahmood, and J. K. Hu. 2016. "A survey of network anomaly detection techniques." *Journal of Network and Computer Applications* 60:19-31. doi: 10.1016/j.jnca.2015.11.016.

Al-Jarrah, O. Y., O. Alhussein, P. D. Yoo, S. Muhaidat, K. Taha, and K. Kim. 2016. "Data Randomization and Cluster-Based Partitioning for Botnet Intrusion Detection." *IEEE Transactions on Cybernetics* 46 (8):1796-1806. doi: 10.1109/TCYB.2015.2490802.

Al-Mhiqani, M. N., R. Ahmad, Z. Z. Abidin, W. Yassin, A. Hassan, K. H. Abdulkareem, N. S. Ali, and Z. Yunos. 2020. "A Review of Insider Threat Detection: Classification, Machine Learning Techniques, Datasets, Open Challenges, and Recommendations." *Applied Sciences-Basel* 10 (15):41. doi: 10.3390/app10155208.

Al-Omari, M., M. Rawashdeh, F. Qutaishat, M. Alshira'H, and N. Ababneh. 2021. "An Intelligent Tree-Based Intrusion Detection Model for Cyber Security." *Journal of Network and Systems Management* 29 (2):18. doi: 10.1007/s10922-021-09591-y.

Alabdallah, A., and M. Awad. 2018. "Using weighted Support Vector Machine to address the imbalanced classes problem of Intrusion Detection System." *Ksii Transactions on Internet and Information Systems* 12 (10):5143-5158. doi: 10.3837/tiis.2018.10.027.

Alazab, M., M. Alazab, A. Shalaginov, A. Mesleh, and A. Awajan. 2020. "Intelligent mobile malware detection using permission requests and API calls." *Future Generation Computer Systems-the International Journal of Escience* 107:509-521. doi: 10.1016/j.future.2020.02.002.

Albahar, M. A., R. A. Al-Falluji, and M. Binsawad. 2020. "An Empirical Comparison on Malicious Activity Detection Using Different Neural Network-Based Models." *IEEE Access* 8:61549-61564. doi: 10.1109/ACCESS.2020.2984157.

AlEroud, A. F., and G. Karabatis. 2018. "Queryable Semantics to Detect Cyber-Attacks: A Flow-Based Detection Approach." *IEEE Transactions on Systems, Man, and Cybernetics: Systems* 48 (2):207-223. doi: 10.1109/TSMC.2016.2600405.

Algarni, A. M., V. Thayananthan, and Y. K. Malaiya. 2021. "Quantitative assessment of cybersecurity risks for mitigating data breaches in business systems." *Applied Sciences (Switzerland)* 11 (8). doi: 10.3390/app11083678.

Alhowaide, A., I. Alsmadi, and J. Tang. 2021. "Towards the design of real-time autonomous IoT NIDS." *Cluster Computing-the Journal of Networks Software Tools and Applications*:14. doi: 10.1007/s10586-021-03231-5.

Ali, S., and Y. Li. 2019. "Learning Multilevel Auto-Encoders for DDoS Attack Detection in Smart Grid Network." *IEEE Access* 7:108647-108659. doi: 10.1109/ACCESS.2019.2933304.

AlKadi, O., N. Moustafa, B. Turnbull, and K. K. R. Choo. 2019. "Mixture Localization-Based Outliers Models for securing Data Migration in Cloud Centers." *IEEE Access* 7:114607-114618. doi: 10.1109/ACCESS.2019.2935142.

Alsamiri, J., and K. Alsubhi. 2019. "Internet of Things Cyber Attacks Detection using Machine Learning." *International Journal of Advanced Computer Science and Applications* 10 (12):627-634.

Alsharafat, W. 2013. "Applying Artificial Neural Network and eXtended Classifier System for Network Intrusion Detection." *International Arab Journal of Information Technology* 10 (3):230-238.

Arcuri, M. C., L. Z. Gai, F. Ielasi, and E. Ventisette. 2020. "Cyber attacks on hospitality sector: stock market reaction." *Journal of Hospitality and Tourism Technology* 11 (2):277-290. doi: 10.1108/jhtt-05-2019-0080.

Ashtiani, M., and M. A. Azgomi. 2014. "A distributed simulation framework for modeling cyber attacks and the evaluation of security measures." *Simulation-Transactions of the Society for Modeling and Simulation International* 90 (9):1071-1102. doi: 10.1177/0037549714540221.

Atefinia, R., and M. Ahmadi. 2021. "Network intrusion detection using multi-architectural modular deep neural network." *Journal of Supercomputing* 77 (4):3571-3593. doi: 10.1007/s11227-020-03410-y.

Avila, R., R. Khoury, R. Khoury, and F. Petrillo. 2021. "Use of Security Logs for Data Leak Detection: A Systematic Literature Review." *Security and Communication Networks* 2021:29. doi: 10.1155/2021/6615899.

Azeez, N. A., T. J. Ayemobola, S. Misra, R. Maskeliunas, and R. Damasevicius. 2019. "Network Intrusion Detection with a Hashing Based Apriori Algorithm Using Hadoop MapReduce." *Computers* 8 (4):15. doi: 10.3390/computers8040086.

Bakdash, J. Z., S. Hutchinson, E. G. Zaroukian, L. R. Marusich, S. Thirumuruganathan, C. Sample, B. Hoffman, and G. Das. 2018. "Malware in the future? Forecasting of analyst detection of cyber events." *Journal of Cybersecurity* 4 (1). doi: 10.1093/cybsec/tyy007.

Barletta, V. S., D. Caivano, A. Nannavecchia, and M. Scalera. 2020. "Intrusion detection for in-vehicle communication networks: An unsupervised kohonen SOM approach." *Future Internet* 12 (7). doi: 10.3390/FI12070119.

Barzegar, M., and M. Shajari. 2018. "Attack scenario reconstruction using intrusion semantics." *Expert Systems with Applications* 108:119-133. doi: 10.1016/j.eswa.2018.04.030.

Bessy-Roland, Yannick, Alexandre Boumezoued, and Caroline Hillairet. 2021. "Multivariate Hawkes process for cyber insurance." *Annals of Actuarial Science* 15 (1):14-39.

Bhardwaj, A., V. Mangat, and R. Vig. 2020. "Hyperband Tuned Deep Neural Network With Well Posed Stacked Sparse AutoEncoder for Detection of DDoS Attacks in Cloud." *IEEE Access* 8:181916-181929. doi: 10.1109/ACCESS.2020.3028690.

Bhati, B. S., C. S. Rai, B. Balamurugan, and F. Al-Turjman. 2020. "An intrusion detection scheme based on the ensemble of discriminant classifiers." *Computers & Electrical Engineering* 86:9. doi: 10.1016/j.compeleceng.2020.106742.

Bhattacharya, S., S. S. R. Krishnan, P. K. R. Maddikunta, R. Kaluri, S. Singh, T. R. Gadekallu, M. Alazab, and U. Tariq. 2020. "A Novel PCA-Firefly Based XGBoost Classification Model for Intrusion Detection in Networks Using GPU." *Electronics* 9 (2):16. doi: 10.3390/electronics9020219.

Bibi, I., A. Akhunzada, J. Malik, J. Iqbal, A. Musaddiq, and S. Kim. 2020. "A Dynamic DL-Driven Architecture to Combat Sophisticated Android Malware." *IEEE Access* 8:129600-129612. doi: 10.1109/ACCESS.2020.3009819.

Biener, C., M. Eling, and J. H. Wirfs. 2015. "Insurability of cyber risk: An empirical analysis." *Geneva Papers on Risk and Insurance: Issues and Practice* 40 (1):131-158. doi: 10.1057/gpp.2014.19.

Binbusayyis, A., and T. Vaiyapuri. 2019. "Identifying and Benchmarking Key Features for Cyber Intrusion Detection: An Ensemble Approach." *IEEE Access* 7:106495-106513. doi: 10.1109/ACCESS.2019.2929487.

Biswas, R., and S. Roy. 2021. "Botnet traffic identification using neural networks." *Multimedia Tools and Applications*:25. doi: 10.1007/s11042-021-10765-8.

Bouyeddou, B., F. Harrou, B. Kadri, and Y. Sun. 2021. "Detecting network cyber-attacks using an integrated statistical approach." *Cluster Computing-the Journal of Networks Software Tools and Applications* 24 (2):1435-1453. doi: 10.1007/s10586-020-03203-1.

Bozkir, A. S., and M. Aydos. 2020. "LogoSENSE: A companion HOG based logo detection scheme for phishing web page and E-mail brand recognition." *Computers & Security* 95:18. doi: 10.1016/j.cose.2020.101855.

Cai, H., F. Zhang, and A. Levi. 2019. "An Unsupervised Method for Detecting Shilling Attacks in Recommender Systems by Mining Item Relationship and Identifying Target Items." *The Computer Journal* 62 (4):579-597. doi: 10.1093/comjnl/bxy124.

Chadza, T., K. G. Kyriakopoulos, and S. Lambotharan. 2020. "Learning to Learn Sequential Network Attacks Using Hidden Markov Models." *IEEE Access* 8:134480-134497. doi: 10.1109/ACCESS.2020.3011293.

Chattopadhyay, M., R. Sen, and S. Gupta. 2018. "A Comprehensive Review and Meta-Analysis on Applications of Machine Learning Techniques in Intrusion Detection." *Australasian Journal of Information Systems* 22:27.

Chen, H. S., and J. Fiscus. 2018. "The inhospitable vulnerability: A need for cybersecurity risk assessment in the hospitality industry." *Journal of Hospitality and Tourism Technology* 9 (2):223-234. doi: 10.1108/JHTT-07-2017-0044.

Chhabra, G. S., V. P. Singh, and M. Singh. 2020. "Cyber forensics framework for big data analytics in IoT environment using machine learning." *Multimedia Tools and Applications* 79 (23-24):15881-15900. doi: 10.1007/s11042-018-6338-1.

Chiba, Z., N. Abghour, K. Moussaid, A. El omri, and M. Rida. 2019. "Intelligent approach to build a Deep Neural Network based IDS for cloud environment using combination of machine learning algorithms." *Computers and Security* 86:291-317. doi: 10.1016/j.cose.2019.06.013.

Choras, M., and R. Kozik. 2015. "Machine learning techniques applied to detect cyber attacks on web applications." *Logic Journal of the Igpl* 23 (1):45-56. doi: 10.1093/jigpal/jzu038.

Chowdhury, Sudipta, Mojtaba Khanzadeh, Ravi Akula, Fangyan Zhang, Song Zhang, Hugh Medal, Mohammad Marufuzzaman, and Linkan Bian. 2017. "Botnet detection using graph-based feature clustering." *Journal of Big Data* 4 (1):14. doi: 10.1186/s40537-017-0074-7.

D'Hooge, L., T. Wauters, B. Volckaert, and F. De Turck. 2019. "Classification Hardness for Supervised Learners on 20 Years of Intrusion Detection Data." *Ieee Access* 7:167455-167469. doi: 10.1109/access.2019.2953451.

Damasevicius, R., A. Venckauskas, S. Grigaliunas, J. Toldinas, N. Morkevicius, T. Aleliunas, and P. Smuikys. 2020. "LITNET-2020: An Annotated Real-World Network Flow Dataset for Network Intrusion Detection." *Electronics* 9 (5):23. doi: 10.3390/electronics9050800.

De Giovanni, Domenico, Arturo Leccadito, and Marco Pirra. 2020. "On the determinants of data breaches: A cointegration analysis." *Decisions in Economics and Finance*. doi: 10.1007/s10203-020-00301-y.

Deng, Lianbing, Daming Li, Xiang Yao, and Haoxiang Wang. 2019. "RETRACTED ARTICLE: Mobile network intrusion detection for IoT system based on transfer learning algorithm." *Cluster Computing* 22 (4):9889-9904. doi: 10.1007/s10586-018-1847-2.

Donkal, G., and G. K. Verma. 2018. "A multimodal fusion based framework to reinforce IDS for securing Big Data environment using Spark." *Journal of Information Security and Applications* 43:1-11. doi: 10.1016/j.jisa.2018.10.001.

Dunn, C., N. Moustafa, and B. Turnbull. 2020. "Robustness Evaluations of Sustainable Machine Learning Models against Data Poisoning Attacks in the Internet of Things." *Sustainability* 12 (16):17. doi: 10.3390/su12166434.

Dwivedi, S., M. Vardhan, and S. Tripathi. 2021. "Multi-Parallel Adaptive Grasshopper Optimization Technique for Detecting Anonymous Attacks in Wireless Networks." *Wireless Personal Communications*:30. doi: 10.1007/s11277-021-08368-5.

Elijah, A. V., A. Abdullah, N. Z. JhanJhi, M. Supramaniam, and O. B. Abdullateef. 2019. "Ensemble and Deep-Learning Methods for Two-Class and Multi-Attack Anomaly Intrusion Detection: An Empirical Study." *International Journal of Advanced Computer Science and Applications* 10 (9):520-528.

Eling, M., and K. Jung. 2018. "Copula approaches for modeling cross-sectional dependence of data breach losses." *Insurance Mathematics & Economics* 82:167-180. doi: 10.1016/j.insmatheco.2018.07.003.

Eling, M., and J. Wirfs. 2019. "What are the actual costs of cyber risk events?" *European Journal of Operational Research* 272 (3):1109-1119. doi: 10.1016/j.ejor.2018.07.021.

Elmasry, W., A. Akbulut, and A. H. Zaim. 2019. "Empirical study on multiclass classification-based network intrusion detection." *Computational Intelligence* 35 (4):919-954. doi: 10.1111/coin.12220.

Elsaid, Shaimaa Ahmed, and Nouf Saleh Albatati. 2020. "An optimized collaborative intrusion detection system for wireless sensor networks." *Soft Computing* 24 (16):12553-12567. doi: 10.1007/s00500-020-04695-0.

Estepa, R., J. E. Díaz-Verdejo, A. Estepa, and G. Madinabeitia. 2020. "How Much Training Data is Enough? A Case Study for HTTP Anomaly-Based Intrusion Detection." *IEEE Access* 8:44410-44425. doi: 10.1109/ACCESS.2020.2977591.

Fan, Z. J., Z. P. Tan, C. X. Tan, and X. Li. 2018. "An Improved Integrated Prediction Method of Cyber Security Situation Based on Spatial-time Analysis." *Journal of Internet Technology* 19 (6):1789-1800. doi: 10.3966/160792642018111906015.

Fang, Z. J., M. C. Xu, S. H. Xu, and T. Z. Hu. 2021. "A Framework for Predicting Data Breach Risk: Leveraging Dependence to Cope With Sparsity." *Ieee Transactions on Information Forensics and Security* 16:2186-2201. doi: 10.1109/tifs.2021.3051804.

Farkas, S., O. Lopez, and M. Thomas. 2021. "Cyber claim analysis using Generalized Pareto regression trees with applications to insurance." *Insurance: Mathematics and Economics* 98:92-105. doi: 10.1016/j.insmatheco.2021.02.009.

Farsi, H., A. Fanian, and Z. Taghiyarrenani. 2019. "A novel online state-based anomaly detection system for process control networks." *International Journal of Critical Infrastructure Protection* 27:11. doi: 10.1016/j.ijcip.2019.100323.

Ferrag, M. A., L. Maglaras, S. Moschoyiannis, and H. Janicke. 2020. "Deep learning for cyber security intrusion detection: Approaches, datasets, and comparative study." *Journal of Information Security and Applications* 50:19. doi: 10.1016/j.jisa.2019.102419.

Fossaceca, J. M., T. A. Mazzuchi, and S. Sarkani. 2015. "MARK-ELM: Application of a novel Multiple Kernel Learning framework for improving the robustness of Network Intrusion Detection." *Expert Systems with Applications* 42 (8):4062-4080. doi: 10.1016/j.eswa.2014.12.040.

Ganeshan, R., and Paul Rodrigues. 2020. "Crow-AFL: Crow Based Adaptive Fractional Lion Optimization Approach for the Intrusion Detection." *Wireless Personal Communications* 111 (4):2065-2089. doi: 10.1007/s11277-019-06972-0.

Gauthama Raman, M. R., Nivethitha Somu, Sahruday Jagarapu, Tina Manghnani, Thirumaran Selvam, Kannan Krithivasan, and V. S. Shankar Sriram. 2020. "An efficient intrusion detection technique based on support vector machine and improved binary gravitational search algorithm." *Artificial Intelligence Review* 53 (5):3255-3286. doi: 10.1007/s10462-019-09762-z.

Gavel, S., A. S. Raghuvanshi, and S. Tiwari. 2021. "Distributed intrusion detection scheme using dual-axis dimensionality reduction for Internet of things (IoT)." *Journal of Supercomputing*:24. doi: 10.1007/s11227-021-03697-5.

Georgescu, T. M., B. Iancu, and M. Zurini. 2019. "Named-entity-recognition-based automated system for diagnosing cybersecurity situations in IoT networks." *Sensors (Switzerland)* 19 (15). doi: 10.3390/s19153380.

Giudici, Paolo, and Emanuela Raffinetti. 2020. "Cyber risk ordering with rank-based statistical models." *AStA Advances in Statistical Analysis*. doi: 10.1007/s10182-020-00387-0.

Goh, Jonathan, Sridhar Adepu, K. N. Junejo, and A. Mathur. 2016. "A Dataset to Support Research in the Design of Secure Water Treatment Systems." CRITIS.

Gong, X. Y., J. L. Lu, Y. F. Zhou, H. Qiu, and R. He. 2021. "Model Uncertainty Based Annotation Error Fixing for Web Attack Detection." *Journal of Signal Processing Systems for Signal Image and Video Technology* 93 (2-3):187-199. doi: 10.1007/s11265-019-01494-1.

Guo, H., S. Huang, C. Huang, Z. Pan, M. Zhang, and F. Shi. 2020. "File Entropy Signal Analysis Combined With Wavelet Decomposition for Malware Classification." *IEEE Access* 8:158961-158971. doi: 10.1109/ACCESS.2020.3020330.

Habib, Maria, Ibrahim Aljarah, and Hossam Faris. 2020. "A Modified Multi-objective Particle Swarm Optimizer-Based Lévy Flight: An Approach Toward Intrusion Detection in Internet of Things." *Arabian Journal for Science and Engineering* 45 (8):6081-6108. doi: 10.1007/s13369-020-04476-9.

Hajj, S., R. El Sibai, J. B. Abdo, J. Demerjian, A. Makhoul, and C. Guyeux. 2021. "Anomaly-based intrusion detection systems: The requirements, methods, measurements, and datasets." *Transactions on Emerging Telecommunications Technologies* 32 (4):36. doi: 10.1002/ett.4240.

Hindy, H., D. Brosset, E. Bayne, A. K. Seeam, C. Tachtatzis, R. Atkinson, and X. Bellekens. 2020. "A Taxonomy of Network Threats and the Effect of Current Datasets on Intrusion Detection Systems." *IEEE Access* 8:104650-104675. doi: 10.1109/ACCESS.2020.3000179.

Hong, W., D. Huang, C. Chen, and J. Lee. 2020. "Towards Accurate and Efficient Classification of Power System Contingencies and Cyber-Attacks Using Recurrent Neural Networks." *IEEE Access* 8:123297-123309. doi: 10.1109/ACCESS.2020.3007609.

Husák, Martin, M. Zádník, V. Bartos, and P. Sokol. 2020. "Dataset of intrusion detection alerts from a sharing platform." *Data in Brief* 33.

Jaber, A. N., and S. Ul Rehman. 2020. "FCM-SVM based intrusion detection system for cloud computing environment." *Cluster Computing-the Journal of Networks Software Tools and Applications* 23 (4):3221-3231. doi: 10.1007/s10586-020-03082-6.

Jahromi, A. N., S. Hashemi, A. Dehghantanha, R. M. Parizi, and K. K. R. Choo. 2020. "An Enhanced Stacked LSTM Method with No Random Initialization for Malware Threat Hunting in Safety and Time-Critical Systems." *IEEE Transactions on Emerging Topics in Computational Intelligence* 4 (5):630-640. doi: 10.1109/TETCI.2019.2910243.

Jang, S., S. Li, and Y. Sung. 2020. "FastText-Based Local Feature Visualization Algorithm for Merged Image-Based Malware Classification Framework for Cyber Security and Cyber Defense." *Mathematics* 8 (3):13. doi: 10.3390/math8030460.

Javeed, D., T. H. Gao, and M. T. Khan. 2021. "SDN-Enabled Hybrid DL-Driven Framework for the Detection of Emerging Cyber Threats in IoT." *Electronics* 10 (8):16. doi: 10.3390/electronics10080918.

Johnson, P., D. Gorton, R. Lagerstrom, and M. Ekstedt. 2016. "Time between vulnerability disclosures: A measure of software product vulnerability." *Computers & Security* 62:278-295. doi: 10.1016/j.cose.2016.08.004.

Johnson, P., R. Lagerström, M. Ekstedt, and U. Franke. 2018. "Can the Common Vulnerability Scoring System be Trusted? A Bayesian Analysis." *IEEE Transactions on Dependable and Secure Computing* 15 (6):1002-1015. doi: 10.1109/TDSC.2016.2644614.

Junger, Marianne, Victoria Wang, and Marleen Schlömer. 2020. "Fraud against businesses both online and offline: crime scripts, business characteristics, efforts, and benefits." *Crime Science* 9 (1):13. doi: 10.1186/s40163-020-00119-4.

Kalutarage, Harsha Kumara, Hoang Nga Nguyen, and Siraj Ahmed Shaikh. 2017. "Towards a threat assessment framework for apps collusion." *Telecommunication Systems* 66 (3):417-430. doi: 10.1007/s11235-017-0296-1.

Kasongo, S. M., and Y. X. Sun. 2020. "A deep learning method with wrapper based feature extraction for wireless intrusion detection system." *Computers & Security* 92:15. doi: 10.1016/j.cose.2020.101752.

Keserwani, Pankaj Kumar, Mahesh Chandra Govil, Emmanuel S. Pilli, and Prajjval Govil. 2021. "A smart anomaly-based intrusion detection system for the Internet of Things (IoT) network using GWO–PSO–RF model." *Journal of Reliable Intelligent Environments* 7 (1):3-21. doi: 10.1007/s40860-020-00126-x.

Keshk, M., E. Sitnikova, N. Moustafa, J. Hu, and I. Khalil. 2021. "An Integrated Framework for Privacy-Preserving Based Anomaly Detection for Cyber-Physical Systems." *IEEE Transactions on Sustainable Computing* 6 (1):66-79. doi: 10.1109/TSUSC.2019.2906657.

Khan, I. A., D. C. Pi, A. K. Bhatia, N. Khan, W. Haider, and A. Wahab. 2020. "Generating realistic IoT-based IDS dataset centred on fuzzy qualitative modelling for cyber-physical systems." *Electronics Letters* 56 (9):441-443. doi: 10.1049/el.2019.4158.

Khraisat, A., I. Gondal, P. Vamplew, J. Kamruzzaman, and A. Alazab. 2020. "Hybrid Intrusion Detection System Based on the Stacking Ensemble of C5 Decision Tree Classifier and One Class Support Vector Machine." *Electronics* 9 (1):18. doi: 10.3390/electronics9010173.

Kilincer, I. F., F. Ertam, and A. Sengur. 2021. "Machine learning methods for cyber security intrusion detection: Datasets and comparative study." *Computer Networks* 188:16. doi: 10.1016/j.comnet.2021.107840.

Kim, D., and H. K. Kim. 2019. "Automated Dataset Generation System for Collaborative Research of Cyber Threat Analysis." *Security and Communication Networks* 2019:10. doi: 10.1155/2019/6268476.

Kirubavathi, G., and R. Anitha. 2016. "Botnet detection via mining of traffic flow characteristics." *Computers & Electrical Engineering* 50:91-101. doi: 10.1016/j.compeleceng.2016.01.012.

Kiwia, D., A. Dehghantanha, K. K. R. Choo, and J. Slaughter. 2018. "A cyber kill chain based taxonomy of banking Trojans for evolutionary computational intelligence." *Journal of Computational Science* 27:394-409. doi: 10.1016/j.jocs.2017.10.020.

Koroniotis, N., N. Moustafa, and E. Sitnikova. 2020. "A new network forensic framework based on deep learning for Internet of Things networks: A particle deep framework." *Future Generation Computer Systems* 110:91-106. doi: 10.1016/j.future.2020.03.042.

Kumar, R., P. Kumar, R. Tripathi, G. P. Gupta, T. R. Gadekallu, and G. Srivastava. 2021. "SP2F: A secured privacy-preserving framework for smart agricultural Unmanned Aerial Vehicles." *Computer Networks* 187. doi: 10.1016/j.comnet.2021.107819.

Kumar, R., and R. Tripathi. 2021. "DBTP2SF: A deep blockchain-based trustworthy privacy-preserving secured framework in industrial internet of things systems." *Transactions on Emerging Telecommunications Technologies* 32 (4):27. doi: 10.1002/ett.4222.

Laso, P. M., D. Brosset, and J. Puentes. 2017. "Dataset of anomalies and malicious acts in a cyber-physical subsystem." *Data in Brief* 14:186-191. doi: 10.1016/j.dib.2017.07.038.

Lee, J., J. Kim, I. Kim, and K. Han. 2019. "Cyber Threat Detection Based on Artificial Neural Networks Using Event Profiles." *IEEE Access* 7:165607-165626. doi: 10.1109/ACCESS.2019.2953095.

Lee, S. J., P. D. Yoo, A. T. Asyhari, Y. Jhi, L. Chermak, C. Y. Yeun, and K. Taha. 2020. "IMPACT: Impersonation Attack Detection via Edge Computing Using Deep Autoencoder and Feature Abstraction." *IEEE Access* 8:65520-65529. doi: 10.1109/ACCESS.2020.2985089.

Levi, M. 2017. "Assessing the trends, scale and nature of economic cybercrimes: overview and Issues: In Cybercrimes, Cybercriminals and Their Policing, in Crime, Law and Social Change." *Crime, Law and Social Change* 67 (1):3-20. doi: 10.1007/s10611-016-9645-3.

Li, D. Q., and Q. M. Li. 2020. "Adversarial Deep Ensemble: Evasion Attacks and Defenses for Malware Detection." *Ieee Transactions on Information Forensics and Security* 15:3886-3900. doi: 10.1109/tifs.2020.3003571.

Li, R. H., C. Zhang, C. Feng, X. Zhang, and C. J. Tang. 2019. "Locating Vulnerability in Binaries Using Deep Neural Networks." *Ieee Access* 7:134660-134676. doi: 10.1109/access.2019.2942043.

Li, X., M. Xu, P. Vijayakumar, N. Kumar, and X. Liu. 2020. "Detection of Low-Frequency and Multi-Stage Attacks in Industrial Internet of Things." *IEEE Transactions on Vehicular Technology* 69 (8):8820-8831. doi: 10.1109/TVT.2020.2995133.

Liu, H. Y., and B. Lang. 2019. "Machine Learning and Deep Learning Methods for Intrusion Detection Systems: A Survey." *Applied Sciences-Basel* 9 (20):28. doi: 10.3390/app9204396.

Lopez-Martin, M., B. Carro, and A. Sanchez-Esguevillas. 2020. "Application of deep reinforcement learning to intrusion detection for supervised problems." *Expert Systems with Applications* 141. doi: 10.1016/j.eswa.2019.112963.

Luo, C. C., S. Su, Y. B. Sun, Q. J. Tan, M. Han, and Z. H. Tian. 2020. "A Convolution-Based System for Malicious URLs Detection." *Cmc-Computers Materials & Continua* 62 (1):399-411. doi: 10.32604/cmc.2020.06507.

Mahbooba, B., M. Timilsina, R. Sahal, and M. Serrano. 2021. "Explainable Artificial Intelligence (XAI) to Enhance Trust Management in Intrusion Detection Systems Using Decision Tree Model." *Complexity* 2021:11. doi: 10.1155/2021/6634811.

Mahdavifar, S., and A. A. Ghorbani. 2020. "DeNNeS: deep embedded neural network expert system for detecting cyber attacks." *Neural Computing & Applications* 32 (18):14753-14780. doi: 10.1007/s00521-020-04830-w.

Mahfouz, A., A. Abuhussein, D. Venugopal, and S. Shiva. 2020. "Ensemble classifiers for network intrusion detection using a novel network attack dataset." *Future Internet* 12 (11):1-19. doi: 10.3390/fi12110180.

Malik, J., A. Akhunzada, I. Bibi, M. Imran, A. Musaddiq, and S. W. Kim. 2020. "Hybrid Deep Learning: An Efficient Reconnaissance and Surveillance Detection Mechanism in SDN." *IEEE Access* 8:134695-134706. doi: 10.1109/ACCESS.2020.3009849.

Manimurugan, S. 2020. "IoT-Fog-Cloud model for anomaly detection using improved Naive Bayes and principal component analysis." *Journal of Ambient Intelligence and Humanized Computing*:10. doi: 10.1007/s12652-020-02723-3.

Martin, A., R. Lara-Cabrera, and D. Camacho. 2019. "Android malware detection through hybrid features fusion and ensemble classifiers: The AndroPyTool framework and the OmniDroid dataset." *Information Fusion* 52:128-142. doi: 10.1016/j.inffus.2018.12.006.

Mauro, M. D., G. Galatro, and A. Liotta. 2020. "Experimental Review of Neural-Based Approaches for Network Intrusion Management." *IEEE Transactions on Network and Service Management* 17 (4):2480-2495. doi: 10.1109/TNSM.2020.3024225.

McLeod, A., and D. Dolezel. 2018. "Cyber-analytics: Modeling factors associated with healthcare data breaches." *Decision Support Systems* 108:57-68. doi: 10.1016/j.dss.2018.02.007.

Miao, Y., J. Ma, X. Liu, J. Weng, H. Li, and H. Li. 2019. "Lightweight Fine-Grained Search Over Encrypted Data in Fog Computing." *IEEE Transactions on Services Computing* 12 (5):772-785. doi: 10.1109/TSC.2018.2823309.

Mireles, J. D., E. Ficke, J. H. Cho, P. Hurley, and S. H. Xu. 2019. "Metrics Towards Measuring Cyber Agility." *Ieee Transactions on Information Forensics and Security* 14 (12):3217-3232. doi: 10.1109/tifs.2019.2912551.

Mishra, N., and S. Pandya. 2021. "Internet of Things Applications, Security Challenges, Attacks, Intrusion Detection, and Future Visions: A Systematic Review." *IEEE Access*. doi: 10.1109/ACCESS.2021.3073408.

Monshizadeh, M., V. Khatri, B. G. Atli, R. Kantola, and Z. Yan. 2019. "Performance Evaluation of a Combined Anomaly Detection Platform." *IEEE Access* 7:100964-100978. doi: 10.1109/ACCESS.2019.2930832.

Moreno, V. C., G. Reniers, E. Salzano, and V. Cozzani. 2018. "Analysis of physical and cyber security-related events in the chemical and process industry." *Process Safety and Environmental Protection* 116:621-631. doi: 10.1016/j.psep.2018.03.026.

Moro, E. D. 2020. "Towards an economic cyber loss index for parametric cover based on IT security indicator: A preliminary analysis." *Risks* 8 (2). doi: 10.3390/risks8020045.

Moustafa, N., E. Adi, B. Turnbull, and J. Hu. 2018. "A New Threat Intelligence Scheme for Safeguarding Industry 4.0 Systems." *IEEE Access* 6:32910-32924. doi: 10.1109/ACCESS.2018.2844794.

Moustakidis, S., and P. Karlsson. 2020. "A novel feature extraction methodology using Siamese convolutional neural networks for intrusion detection." *Cybersecurity* 3 (1). doi: 10.1186/s42400-020-00056-4.

Mukhopadhyay, Arunabha, Samir Chatterjee, Kallol K. Bagchi, Peteer J. Kirs, and Girja K. Shukla. 2019. "Cyber Risk Assessment and Mitigation (CRAM) Framework Using Logit and Probit Models for Cyber Insurance." *Information Systems Frontiers* 21 (5):997-1018. doi: 10.1007/s10796-017-9808-5.

Murugesan, V., M. Shalinie, and M. H. Yang. 2018. "Design and analysis of hybrid single packet IP traceback scheme." *Iet Networks* 7 (3):141-151. doi: 10.1049/iet-net.2017.0115.

Mwitondi, K. S., and S. A. Zargari. 2018. "An iterative multiple sampling method for intrusion detection." *Information Security Journal* 27 (4):230-239. doi: 10.1080/19393555.2018.1539790.

Neto, N. N., S. Madnick, A. M. G. De Paula, and N. M. Borges. 2021. "Developing a Global Data Breach Database and the Challenges Encountered." *Acm Journal of Data and Information Quality* 13 (1):33. doi: 10.1145/3439873.

Oliveira, N., I. Praca, E. Maia, and O. Sousa. 2021. "Intelligent Cyber Attack Detection and Classification for Network-Based Intrusion Detection Systems." *Applied Sciences-Basel* 11 (4):21. doi: 10.3390/app11041674.

Pajouh, H. H., R. Javidan, R. Khayami, A. Dehghantanha, and K. R. Choo. 2019. "A Two-Layer Dimension Reduction and Two-Tier Classification Model for Anomaly-Based Intrusion Detection in IoT Backbone Networks." *IEEE Transactions on Emerging Topics in Computing* 7 (2):314-323. doi: 10.1109/TETC.2016.2633228.

Parra, G. D., P. Rad, K. K. R. Choo, and N. Beebe. 2020. "Detecting Internet of Things attacks using distributed deep learning." *Journal of Network and Computer Applications* 163:13. doi: 10.1016/j.jnca.2020.102662.

Paté-Cornell, M. E., M. Kuypers, M. Smith, and P. Keller. 2018. "Cyber Risk Management for Critical Infrastructure: A Risk Analysis Model and Three Case Studies." *Risk Analysis* 38 (2):226-241. doi: 10.1111/risa.12844.

Pooser, David M., Mark J. Browne, and Oleksandra Arkhangelska. 2018. "Growth in the Perception of Cyber Risk: Evidence from U.S. P&C Insurers." *The Geneva Papers on Risk and Insurance - Issues and Practice* 43 (2):208-223. doi: 10.1057/s41288-017-0077-9.

Pu, G., L. Wang, J. Shen, and F. Dong. 2021. "A hybrid unsupervised clustering-based anomaly detection method." *Tsinghua Science and Technology* 26 (2):146-153. doi: 10.26599/TST.2019.9010051.

Qiu, J., W. Luo, L. Pan, Y. Tai, J. Zhang, and Y. Xiang. 2019. "Predicting the Impact of Android Malicious Samples via Machine Learning." *IEEE Access* 7:66304-66316. doi: 10.1109/ACCESS.2019.2914311.

Qu, X., L. Yang, K. Guo, M. Sun, L. Ma, T. Feng, S. Ren, K. Li, and X. Ma. 2020. "Direct Batch Growth Hierarchical Self-Organizing Mapping Based on Statistics for Efficient Network Intrusion Detection." *IEEE Access* 8:42251-42260. doi: 10.1109/ACCESS.2020.2976810.

Rahman, Md Shafiur, Sajal Halder, Md Ashraf Uddin, and Uzzal Kumar Acharjee. 2021. "An efficient hybrid system for anomaly detection in social networks." *Cybersecurity* 4 (1):10. doi: 10.1186/s42400-021-00074-w.

Ramaiah, M., V. Chandrasekaran, V. Ravi, and N. Kumar. 2021. "An intrusion detection system using optimized deep neural network architecture." *Transactions on Emerging Telecommunications Technologies* 32 (4):17. doi: 10.1002/ett.4221.

Raman, M. R. G., K. Kannan, S. K. Pal, and V. S. S. Sriram. 2016. "Rough Set-hypergraph-based Feature Selection Approach for Intrusion Detection Systems." *Defence Science Journal* 66 (6):612-617. doi: 10.14429/dsj.66.10802.

Sarabi, A., P. Naghizadeh, Y. Liu, and M. Liu. 2016. "Risky business: Fine-grained data breach prediction using business profiles." *Journal of Cybersecurity* 2 (1):15-28. doi: 10.1093/cybsec/tyw004.

Sarker, Iqbal H., A. S. M. Kayes, Shahriar Badsha, Hamed Alqahtani, Paul Watters, and Alex Ng. 2020. "Cybersecurity data science: an overview from machine learning perspective." *Journal of Big Data* 7 (1):41. doi: 10.1186/s40537-020-00318-5.

Sentuna, A., A. Alsadoon, P. W. C. Prasad, M. Saadeh, and O. H. Alsadoon. 2021. "A Novel Enhanced Naïve Bayes Posterior Probability (ENBPP) Using Machine Learning: Cyber Threat Analysis." *Neural Processing Letters* 53 (1):177-209. doi: 10.1007/s11063-020-10381-x.

Shaukat, K., S. H. Luo, V. Varadharajan, I. A. Hameed, S. Chen, D. X. Liu, and J. M. Li. 2020. "Performance Comparison and Current Challenges of Using Machine Learning Techniques in Cybersecurity." *Energies* 13 (10):27. doi: 10.3390/en13102509.

Sheehan, B., F. Murphy, M. Mullins, and C. Ryan. 2019. "Connected and autonomous vehicles: A cyber-risk classification framework." *Transportation Research Part A: Policy and Practice* 124:523-536. doi: 10.1016/j.tra.2018.06.033.

Sheehan, Barry, Finbarr Murphy, Arash N. Kia, and Ronan Kiely. 2021. "A quantitative bow-tie cyber risk classification and assessment framework." *Journal of risk research*:1-20.

Shlomo, A., M. Kalech, and R. Moskovitch. 2021. "Temporal pattern-based malicious activity detection in SCADA systems." *Computers & Security* 102:17. doi: 10.1016/j.cose.2020.102153.

Singh, K. J., and T. De. 2020. "Efficient Classification of DDoS Attacks Using an Ensemble Feature Selection Algorithm." *Journal of Intelligent Systems* 29 (1):71-83. doi: 10.1515/jisys-2017-0472.

Skrjanc, I., S. Ozawa, T. Ban, and D. Dovzan. 2018. "Large-scale cyber attacks monitoring using Evolving Cauchy Possibilistic Clustering." *Applied Soft Computing* 62:592-601. doi: 10.1016/j.asoc.2017.11.008.

Sornette, D., T. Maillart, and W. Kröger. 2013. "Exploring the limits of safety analysis in complex technological systems." *International Journal of Disaster Risk Reduction* 6:59-66. doi: 10.1016/j.ijdrr.2013.04.002.

Sovacool, Benjamin K. 2008. "The costs of failure: A preliminary assessment of major energy accidents, 1907–2007." *Energy Policy* 36 (5):1802-1820. doi: <https://doi.org/10.1016/j.enpol.2008.01.040>.

Stojanovic, B., K. Hofer-Schmitz, and U. Kleb. 2020. "APT datasets and attack modeling for automated detection methods: A review." *Computers & Security* 92:19. doi: 10.1016/j.cose.2020.101734.

Subroto, A., and A. Apriyana. 2019. "Cyber risk prediction through social media big data analytics and statistical machine learning." *Journal of Big Data* 6 (1). doi: 10.1186/s40537-019-0216-1.

Tan, Z., A. Jamdagni, X. He, P. Nanda, R. P. Liu, and J. Hu. 2015. "Detection of Denial-of-Service Attacks Based on Computer Vision Techniques." *IEEE Transactions on Computers* 64 (9):2519-2533. doi: 10.1109/TC.2014.2375218.

Uhm, Y., and W. Pak. 2021. "Service-Aware Two-Level Partitioning for Machine Learning-Based Network Intrusion Detection With High Performance and High Scalability." *IEEE Access* 9:6608-6622. doi: 10.1109/ACCESS.2020.3048900.

Ulven, J. B., and G. Wangen. 2021. "A systematic review of cybersecurity risks in higher education." *Future Internet* 13 (2):1-40. doi: 10.3390/fi13020039.

Valeriano, B., and R. C. Maness. 2014. "The dynamics of cyber conflict between rival antagonists, 2001-11." *Journal of Peace Research* 51 (3):347-360. doi: 10.1177/0022343313518940.

Varghese, J. E., and B. Muniyal. 2021. "An Efficient IDS Framework for DDoS Attacks in SDN Environment." *IEEE Access* 9:69680-69699. doi: 10.1109/ACCESS.2021.3078065.

Velliangiri, S., and H. M. Pandey. 2020. "Fuzzy-Taylor-elephant herd optimization inspired Deep Belief Network for DDoS attack detection and comparison with state-of-the-arts algorithms." *Future Generation Computer Systems-the International Journal of Escience* 110:80-90. doi: 10.1016/j.future.2020.03.049.

Verma, A., and V. Ranga. 2020. "Machine Learning Based Intrusion Detection Systems for IoT Applications." *Wireless Personal Communications* 111 (4):2287-2310. doi: 10.1007/s11277-019-06986-8.

Vidros, S., C. Kolias, G. Kambourakis, and L. Akoglu. 2017. "Automatic Detection of Online Recruitment Frauds: Characteristics, Methods, and a Public Dataset." *Future Internet* 9 (1):19. doi: 10.3390/fi9010006.

Vinayakumar, R., M. Alazab, K. P. Soman, P. Poornachandran, A. Al-Nemrat, and S. Venkatraman. 2019. "Deep Learning Approach for Intelligent Intrusion Detection System." *Ieee Access* 7:41525-41550. doi: 10.1109/access.2019.2895334.

Walker-Roberts, S., M. Hammoudeh, O. Aldabbas, M. Aydin, and A. Dehghantanha. 2020. "Threats on the horizon: understanding security threats in the era of cyber-physical systems." *Journal of Supercomputing* 76 (4):2643-2664. doi: 10.1007/s11227-019-03028-9.

Xin, Y., L. Kong, Z. Liu, Y. Chen, Y. Li, H. Zhu, M. Gao, H. Hou, and C. Wang. 2018. "Machine Learning and Deep Learning Methods for Cybersecurity." *IEEE Access* 6:35365-35381. doi: 10.1109/ACCESS.2018.2836950.

Xu, Chang, Jie Zhang, Kuiyu Chang, and Chong Long. 2013. "Uncovering collusive spammers in Chinese review websites." Proceedings of the 22nd ACM international conference on Information & Knowledge Management.

Yang, J., T. Li, G. Liang, W. He, and Y. Zhao. 2019. "A Simple Recurrent Unit Model Based Intrusion Detection System With DCGAN." *IEEE Access* 7:83286-83296. doi: 10.1109/ACCESS.2019.2922692.

Yuan, B. G., J. F. Wang, D. Liu, W. Guo, P. Wu, and X. H. Bao. 2020. "Byte-level malware classification based on markov images and deep learning." *Computers & Security* 92:12. doi: 10.1016/j.cose.2020.101740.

Zhang, S., X. M. Ou, and D. Caragea. 2015. "Predicting Cyber Risks through National Vulnerability Database." *Information Security Journal* 24 (4-6):194-206. doi: 10.1080/19393555.2015.1111961.

Zhou, X., W. Liang, S. Shimizu, J. Ma, and Q. Jin. 2021. "Siamese Neural Network Based Few-Shot Learning for Anomaly Detection in Industrial Cyber-Physical Systems." *IEEE Transactions on Industrial Informatics* 17 (8):5790-5798. doi: 10.1109/TII.2020.3047675.

Zhou, Y. Y., G. Cheng, S. Q. Jiang, and M. Dai. 2020. "Building an efficient intrusion detection system based on feature selection and ensemble classifier." *Computer Networks* 174:17. doi: 10.1016/j.comnet.2020.107247.
